# Approved immune checkpoint inhibitors in hepatocellular carcinoma: a large-scale meta-analysis and systematic review

**DOI:** 10.1007/s00432-023-05539-8

**Published:** 2024-02-06

**Authors:** Ruyi Zhang, Fang Wang, Zhiyu You, Dongyang Deng, Jiangyan He, Wentao Yan, Jian Quan, Jing Wang, Shujuan Yan

**Affiliations:** 1 Department of Clinical Laboratory, First Affiliated Hospital of Guizhou University of Traditional Chinese Medicine, Guizhou, 550001 China; 2 Center for Eugenics Research, First Affiliated Hospital of Guizhou University of Traditional Chinese Medicine, Guiyang, 550001 China; 3 Department of Clinical Laboratory, Anshun Hospital of Guizhou Aviation Industry Group, Guizhou, 561099 China; 4 Department of Orthopedic, Kunming Hospital of Chinese Medicine, Kunming, 650051 China; 5 Department of Prenatal Diagnostic Center, Guangzhou Women and Children’s Medical Centre, Guangzhou Medical University, Tianhe District, No.9 Jinsui Road, Zhujiang New Town, Guangzhou, 510623 People’s Republic of China; 6 Department of Prenatal Diagnosis Center, Guizhou Provincial People’s Hospital, the Affiliated Hospital of Guizhou University, Guiyang, 550000 Guizhou Province China

**Keywords:** Hepatocellular carcinoma, Immunotherapy, Checkpoint inhibitor, Treatment effect, Adverse events

## Abstract

A meta-analysis was performed to assess the benefits and safety profile of approved immune checkpoint inhibitors in hepatocellular carcinoma patients. Eligible studies were searched from Cochrane, Embase, and PubMed databases based on a well-established strategy. Following the exclusion of ineligible studies, 12 studies were included in this meta-analysis. Compared with control group, immune checkpoint inhibitors were associated with improved ORR (OR 3.03, 95% CI 2.26–4.05, *P* < 0.00001), SD (OR 0.77, 95% CI 0.62–0.95, *P* = 0.02), OS (HR 0.75, 95% CI 0.68–0.83, *P* < 0.00001), and PFS (HR 0.74, 95% CI 0.63–0.87, *P* < 0.0003). However, no significant differences were observed in DCR (OR 1.33, 95% CI 0.97–1.81, *P* = 0.07), PD (OR 0.90, 95% CI 0.67–1.21, *P* = 0.48), and all caused any-grade adverse events (OR 1.22, 95% CI 0.62–2.39, *P* = 0. 57), all caused ≥ grade 3 adverse events (OR 1.10, 95% CI 0.97–1.25, *P* = 0.14), treatment-related any-grade adverse events (OR 1.13, 95% CI 0.55–2.32, *P* = 0.73), and treatment-related ≥ grade 3 events (OR 0.82, 95% CI 0.34–1.97, *P* = 0.65) between the two groups. After subgroup analysis conducted, patients in the immune checkpoint inhibitor group compared with targeted drug group showed significant improvements in OS (HR 0.74, 95% CI 0.66–0.84, *P* < 0.00001) and PFS (HR 0.75, 95% CI 0.61–0.91, *P* = 0.004). Immune checkpoint inhibitors have demonstrated peculiar benefits in the treatment of HCC with an acceptable safety profile. Compared to targeted drugs, immune checkpoint inhibitors still offer advantages in the treatment of hepatocellular carcinoma. However, there is still considerable room for further improvement.

**Supplementary Information:**

The online version contains supplementary material available at 10.1007/s00432-023-05539-8.

## Introduction

Hepatocellular carcinoma (HCC) is the most prevalent form of primary liver cancer, constituting more than 90% of all primary liver tumors (Gajos-Michniewicz and Czyz 2023; Konyn et al. 2021; Lee et al. 2022). It represents a significant cause of cancer-related mortality worldwide, with an estimated annual death toll exceeding 800,000 individuals. Alarmingly, the World Health Organization predicts that over the next decade, liver cancer will claim the lives of more than one million individuals (Stefan and Cusi 2022).

Multiple risk factors contribute to the development of HCC, many of which are modifiable. The primary risk factor is the infection of hepatitis B or C virus (HBV or HCV), accounting for approximately 80% of HCC cases globally (Kanwal et al. 2022). The prevalence of these viral infections in developing countries further contributes to the higher burden of liver cancer in these regions. Additionally, other risk factors include excessive alcohol consumption, obesity, cigarette smoking, and exposure to environmental toxins.

In the past decade, the emergence of small molecular targeted drugs has brought a glimmer of hope to patients with hepatocellular carcinoma (HCC) (Chen et al. 2020). Sorafenib, the first drug approved for systemic treatment of advanced HCC, has exhibited modest improvements in median overall survival in phase III clinical trials (Llovet et al. 2018). Lenvatinib, another oral multikinase inhibitor, has demonstrated efficacy against poorly differentiated or highly malignant grade HCC by selectively inhibiting specific receptor tyrosine kinases involved in tumor formation and angiogenesis (Rinaldi et al. 2021). Furthermore, regorafenib, which inhibits multiple protein kinases (VEGFR1-3, TIE2, PDGFRβ, FGFR, KIT, RET, RAF-1, and BRAF) with a molecular structure almost identical to sorafenib, has shown a toxicity profile similar to that of sorafenib, as observed following the addition of a fluorine bond (Xing et al. 2021). In a phase III placebo-controlled clinical trial, the regorafenib group exhibited significantly superior overall survival (OS) compared to the placebo group, along with significantly longer progression-free survival (PFS) and time to progression (TTP) (Bruix et al. 2017). As a second-line therapy, regorafenib became the first drug to demonstrate efficacy after the progression on sorafenib, compared to a placebo. However, one of the major challenges encountered by patients undergoing treatment with these targeted drugs is the development of tumor resistance within a relatively short timeframe (Dai et al. 2022). Advancements in immune checkpoint inhibitors (ICIs) have offered some solace for certain HCC patients, exhibiting efficacy in specific cases and presenting new treatment possibilities beyond traditional approaches (El Dika et al. 2019).

Immune checkpoint inhibitors (ICIs) are monoclonal antibodies that disrupt the interaction between extracellular ligands or receptors, thereby interfering with antitumor immune responses. These proteins are expressed by both the immune system and tumor cells (Liu et al. 2021). Since the initial approval of an immune checkpoint inhibitor for melanoma treatment in March 2011, the scope of indications for ICIs has expanded (Singh et al. 2020). Currently, there are eight ICIs approved globally for the treatment of hepatocellular carcinoma (HCC) (nivolumab, durvalumab, tislelizumab, ipilimumab, pembrolizumab, atezolizumab, tremelimumab, and sintilimab), with the highly probable approval of camrelizumab in the near future. The introduction of these ICIs has contributed to a partial improvement in the survival rates of HCC patients (Donisi et al. 2020; Liu and Qin 2019).

Nivolumab, a human anti-PD-1 (programmed cell death protein 1) IgG4 monoclonal antibody that inhibits PD-1, received US Food and Drug Administration (FDA) approval in 2017 as a second-line therapy for HCC patients who had experienced disease progression after initial sorafenib treatment (Wong et al. 2021). The results of the phase I/II dose-escalation and expansion trial, known as CheckMate 040, which included HCC patients with varying Child–Pugh scores, indicated that nivolumab exhibited a manageable toxicity profile and led to considerable tumor reduction. Another randomized phase III study (CheckMate 459) evaluated the efficacy of nivolumab compared to sorafenib (Yau et al. 2022). However, the objective response rate (ORR) in the nivolumab and sorafenib groups was 15% and 7%, respectively, and overall survival did not meet the pre-defined statistical significance threshold (HR 0.84, P = 0.042) in nivolumab-treated HCC patients.

Atezolizumab, an engineered IgG1 monoclonal antibody-based biotherapeutics, was specifically designed to inhibit programmed death ligand 1 (PD-L1). A randomized phase Ib study demonstrated a manageable adverse reaction profile and significantly improved progression-free survival in HCC patients receiving atezolizumab plus bevacizumab compared to atezolizumab monotherapy (Finn et al. 2020a). However, in a phase II KEYNOTE-224 trial, overall survival and progression-free survival of patients treated with pembrolizumab did not reach statistical significance according to the pre-specified criteria (Zhu et al. 2018).

It is important to acknowledge that the impact of ICIs on HCC has been clearly demonstrated. Several meta-analyses have also been conducted to further elucidate the therapeutic effects of ICIs on HCC from various perspectives (Fong et al. 2023; Kulkarni et al. 2023). However, certain limitations exist within these meta-analyses. Some of them include studies involving unapproved ICIs, which can confound the evaluation of the effectiveness of ICIs that are specifically approved for the treatment of HCC. Consequently, this inclusion of unapproved ICIs may undermine the credibility of the final conclusions. Moreover, some meta-analyses might give the impression of including many studies, but upon closer examination, it becomes evident that among the included studies, some are single-arm trials or limited number of participants. These factors increase the risk of publication bias. Therefore, taking these limitations into consideration, the main objective of our present study is to compare the effectiveness and safety of approved ICIs with that of small molecule drugs or placebos in the treatment of hepatocellular carcinoma. By conducting this investigation, we aim to gain a comprehensive understanding of the current status of ICIs in the management of hepatocellular carcinoma.

## Materials and methods

This meta-analysis and systematic review was performed in accordance with the guidelines of the Cochrane handbook for systematic reviews of interventions and PRISMA (Preferred Reporting Items for Systematic Reviews and Meta-Analysis).

### Data sources and search

Online databases and websites, including Embase, PubMed, and the Cochrane Database, were searched for eligible studies from January 1, 2011 to October 07, 2023. Search terms included “hepatocellular carcinoma” and “nivolumab” or “durvalumab” or “tislelizumab” or “ipilimumab” or “pembrolizumab” or “atezolizumab” or “camrelizumab” or “tremelimumab” or “sintilimab,” and “checkpoint inhib*.” Abstracts, posters, and references in the eligible articles were also searched when necessary. If necessary, relevant studies would seek from the relevant literature (Table S1).

Two investigators, Z.R.Y and F.W, initially reviewed the title and abstract of the retrieved studies. The full article of every study that matched the inclusion criteria was read by 3 individuals, J.Y.H, D.Y.D, and J.Q, to further assess eligibility. If disagreements arose, all the researchers further discussed until a consensus was reached.

### Study selection

Researches were included if they met the following criteria: (1) published in English; (2) ICIs were approved by at least one national agency for use in patients with HCC; (3) population: advanced HCC patients; (4) studies with head-to-head comparisons comprising at least two groups; (5) at least one group of patients was given an immune checkpoint inhibitor, and the control group should not receive an immune checkpoint inhibitor; (6) studies reporting at least one of the following results: objective response rate (ORR), disease control rate (DCR), OS, PFS, and adverse events; And (7) clinical efficacy assessment was based on response evaluation criteria in solid tumors (RECIST), modified RECIST (mRECIST), or immune-related RECIST (iRECIST). The exclusion criteria were as follows: (1) duplicated articles, (2) article type: review articles, case reports, systematic reviews, and meta-analyses, and (3) studies with limited sample size (the study involved fewer than 100 people). If data from two or more studies overlapped, the study with the most recent or more extensive data was selected for our study. It is important to note that while camrelizumab + apatinib is not currently approved for HCC treatment, it is highly likely to receive approval based on existing clinical trial data. As a result, this study included camrelizumab plus apatinib.

### Data extraction

A data extraction form was developed to capture specific study characteristics and clinical data. The form included the following information: primary author, publication year, geographical location, trial name or clinical trial identification number, RECIST version, use of mRECIST, type of inhibitors employed, trial phase, patient count, objective response rate (ORR), disease control rate (DCR), stable disease (SD), progressive disease (PD), progression-free survival (PFS), overall survival (OS), and adverse events.

The quality of the included studies was assessed using RoB2.0 tool and ROBINS-I tool. RoB2.0 tool included 5 dimensionality parameters: bias arising from the randomization process, bias due to deviations from intended intervention, bias due to missing outcome data, bias in measurement of the outcome, and bias in selection of the reported result. Each parameter was categorized as follows: High, Some concerns, Low, and No information.

### Data analysis

The statistical data analyses in this study utilized various software tools including Microsoft Office Excel, the "R" programming language with the packages meta, Matrix, metafor, readr, openxlsx, and Review Manager version 5.4. The quality of the included research was independently evaluated by two investigators (R.Y.Z and S.J.Y). The assignment of patients to the control group involved those treated with molecular targeted drugs or placebo, while patients treated with immune checkpoint inhibitors were assigned to the intervention group.

Statistical calculations included the calculation of 95% confidence intervals and odds ratios (OR) for outcomes such as overall response rate (ORR), disease control rate (DCR), stable disease (SD), progressive disease (PD), and adverse events. Hazard ratios (HRs) were calculated for progression-free survival (PFS) and overall survival (OS). The Mantel–Haenszel method in RevMan software was used for analysis. Heterogeneity for each result was assessed using the I^2^ statistics, with I^2^ values categorized as < 30% (low heterogeneity), 30%–50% (moderate heterogeneity), 50%–75% (substantial heterogeneity), and > 75% (significant heterogeneity). In the presence of substantial heterogeneity, random-effects models were used to calculate OR and 95% confidence intervals. For results without substantial heterogeneity, fixed-effects models were applied. Subgroup analyses were conducted when necessary, particularly in relation to the control group. The specific HR values were derived from the original data of the studies. Publication bias was assessed using funnel plots and Egger's test. The overall quality of evidence was evaluated using the Grading of Recommendations Assessment, Development, and Evaluation (GRADE) approach. Sensitivity analysis was performed using the one-by-one elimination method.

## Results

### Study characteristics

**Fig. 1:**
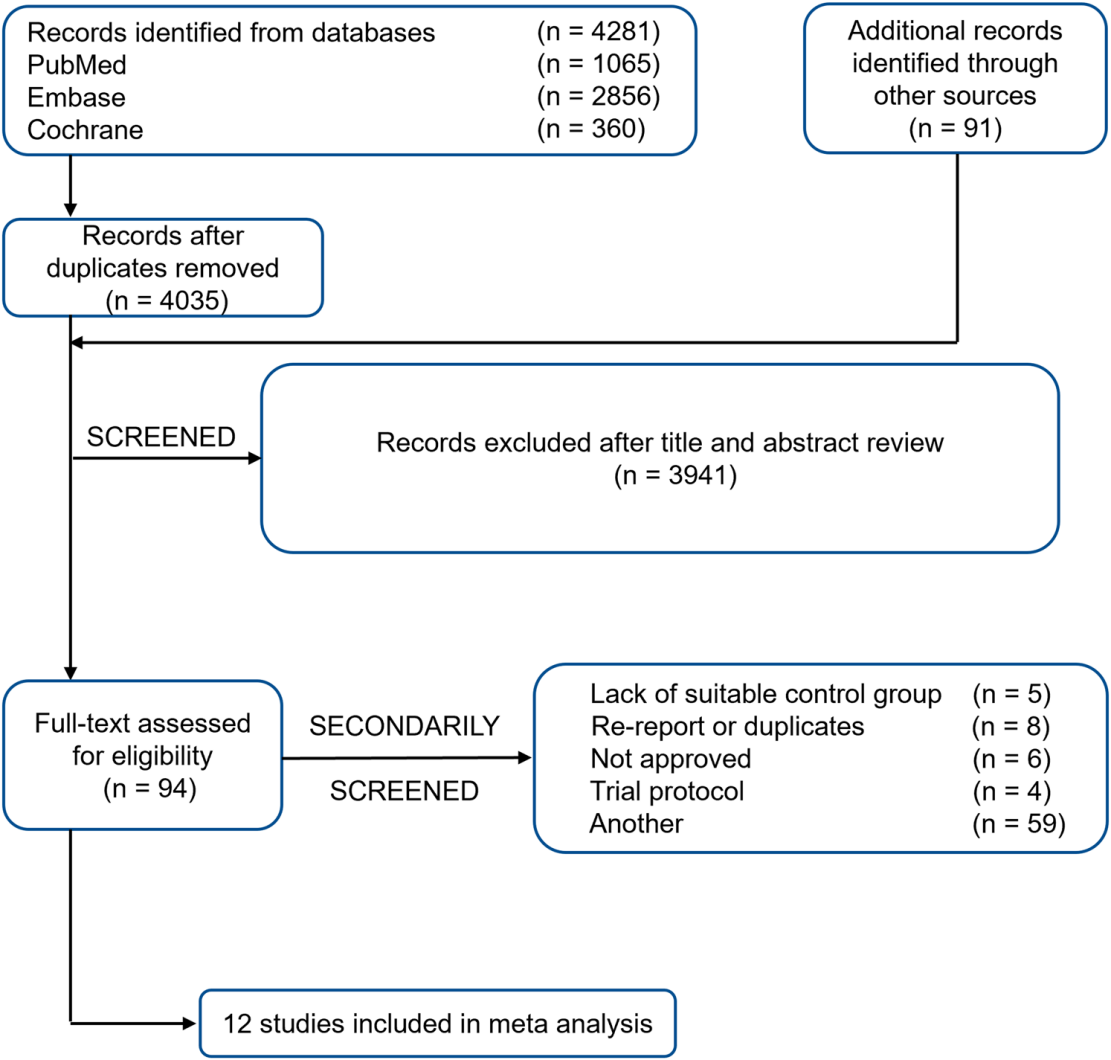
Study flow diagram

A total of 4,372 records were retrieved using the search strategy in the Embase, PubMed, and Cochrane databases, among which 12 papers matched the criteria for inclusion in this meta-analysis (Fig. 1, Table 1). All studies have been published in recent years. Of the 12 studies, 10 studies were phase III clinical trials, and 2 were retrospective cohort study. Ten of the studies were multicenter, global studies involving a multiracial population, while two studies were conducted at a single center, with Asians accounting for most of the participants (Table 1). Robin K. study set up two control groups, one group was sorafenib and the other group was cabozantinib. According to the setting of this study, we subdivided this study into two studies.

**Table 1:** Characteristics of all included studies

Author	Year	Trial/Phase	RECIST Version	Types of clinical studies	Country	Clinical trials ID	Majority Ethnicity	Treatment Arm	PD-1/PD-L1/CTLA4	Control Arm	Pharmaceutical company	NO Patients
^[1]^ Richard S	2020	Imbrave150 /phase III	RECIST 1.1	RCT^*^	Global, multicenter	NCT03434379	Multi-ethnic	Atezolizumab + Bevacizumab	PD-L1	Sorafenib	Roche	501
^[2]^ Richard S	2019	KEYNOTE-240/phase III	RECIST 1.1	RCT	Global, multicenter	NCT02702401	Multi-ethnic	Pembrolizumab	PD-1	Placebo	Merck Sharp& Dohme (MSD)	413
^[3]^ Won M	2020	–	Mrecist	Observational study	Single center	–	Asia	Nivolumab	PD-1	Regorafenib	Bristol-Myers Squibb	373
^[4]^ Cheol H	2020	—	RECIST 1.1	Observational study	Single center	—	Asia	Nivolumab	PD-1	Regorafenib	Bristol-Myers Squibb	105
^[5]^ Yau T	2022	CheckMate 459/phase III	RECIST v1.1	RCT	Global, multicenter	NCT02576509	Multi-ethnic	Nivolumab	PD-1	Sorafenib	Bristol-Myers Squibb	743
^[6]^ Shukui Q. (P)	2023	KEYNOTE-394/phase III	RECIST v1.1	RCT	Multicenter	NCT03062358	Asia	Pembrolizumab	PD-1	Placebo	Merck Sharp& Dohme (MSD)	453
^[7]^ Zhenggang R	2021	ORIENT-32/phase II–III	RECIST v1.1	RCT	Multicenter	NCT03794440	Asia	Sintilimab plus IBI305	PD-1	Sorafenib	Innovent and Lilly	571
^[8]^ Finn R	2022	LEAP-002/phase III	RECIST v1.1	RCT	Global, Multicenter	NCT03713593	Multi-ethnic	Lenvatinib + Pembrolizumab	PD-1	Lenvatinib	Merck Sharp& Dohme (MSD)	794
^[9]^ Robin K.^#^	2022	COSMIC-312/phase III	RECIST v1.1	RCT	Global, Multicenter	NCT03755791	Multi-ethnic	Cabozantinib + Atezolizumab	PD-1	Sorafenib/ Cabozantinib	Hengrui Medicine	837
^[10]^ Ghassan K	2023	HIMALAYA/phase III	RECIST v1.1	RCT	Multicenter	NCT03298451	Multi-ethnic	Tremelimumab plus durvalumab	CTLA-4/PD-L1	Sorafenib	AstraZeneca	1171
^[11]^ Shukui Q. (C)	2023	CARES-310/phase III	RECIST v1.1	RCT	Multicenter	NCT03764293	Asia	Camrelizumab plus apatinib	PD-1	Sorafenib	Hengrui Medicine	543
^[12]^ Shukui Q. (T)	2023	RATIONALE-301/phase III	RECIST v1.1	RCT	Multicenter	NCT03412773	Multi-ethnic	Tislelizumab	PD-1	Sorafenib	BeiGene	674

^#^The control group of this study included two groups of people taking sorafenib and cabozantinib, so we divided them into two groups for investigated. Robin K. (S) represented the control group taking sorafenib. Robin K. (C) represented the control group taking cabozantinib

*: Randomized Controlled Trial

[1] https://doi.org/10.1056/NEJMoa1915745

[2] https://doi.org/10.1200/JCO.19.01307

[3] https://doi.org/10.1002/hep4.1523

[4] https://doi.org/10.3350/cmh.2019.0049n

[5] https://doi.org/10.1016/S1470-2045(21)00604-5

[6] https://doi.org/10.1200/JCO.22.00620

[7] https://doi.org/10.1016/S1470-2045(21)00252-7

[8] https://doi.org/10.1016/annonc/annonc1089

[9] https://doi.org/10.1016/S1470-2045(22)00326-6

[10] https://doi.org/10.1056/EVIDoa2100070

[11] https://doi.org/10.1016/S0140-6736(23)00961-3

[12] https://doi.org/10.1001/jamaoncol.2023.4003

Three types of immune checkpoint inhibitors (PD-1/PD-L1/ CTLA-4) were involved in the research. PD-1 inhibitors (pembrolizumab, sintilimab, and nivolumab) were used in 10 studies, whereas two studies used the PD-L1 inhibitor atezolizumab and durvalumab as the intervention group. Four studies enrolled patients with unresectable hepatocellular carcinoma, while those with sorafenib intolerance or disease progression were enrolled in two studies. The bias risk is attached to the supplementary materials (Figure S1).

To enhance our overall comprehension of the utilization of ICIs in HCC, we have compiled and analyzed the meta-analyses published in recent years that evaluate the influence of ICIs on HCC (predictive or diagnostic meta-analyses were excluded from our compilation). (Table S2).

### ORR and DCR

**Fig. 2:**
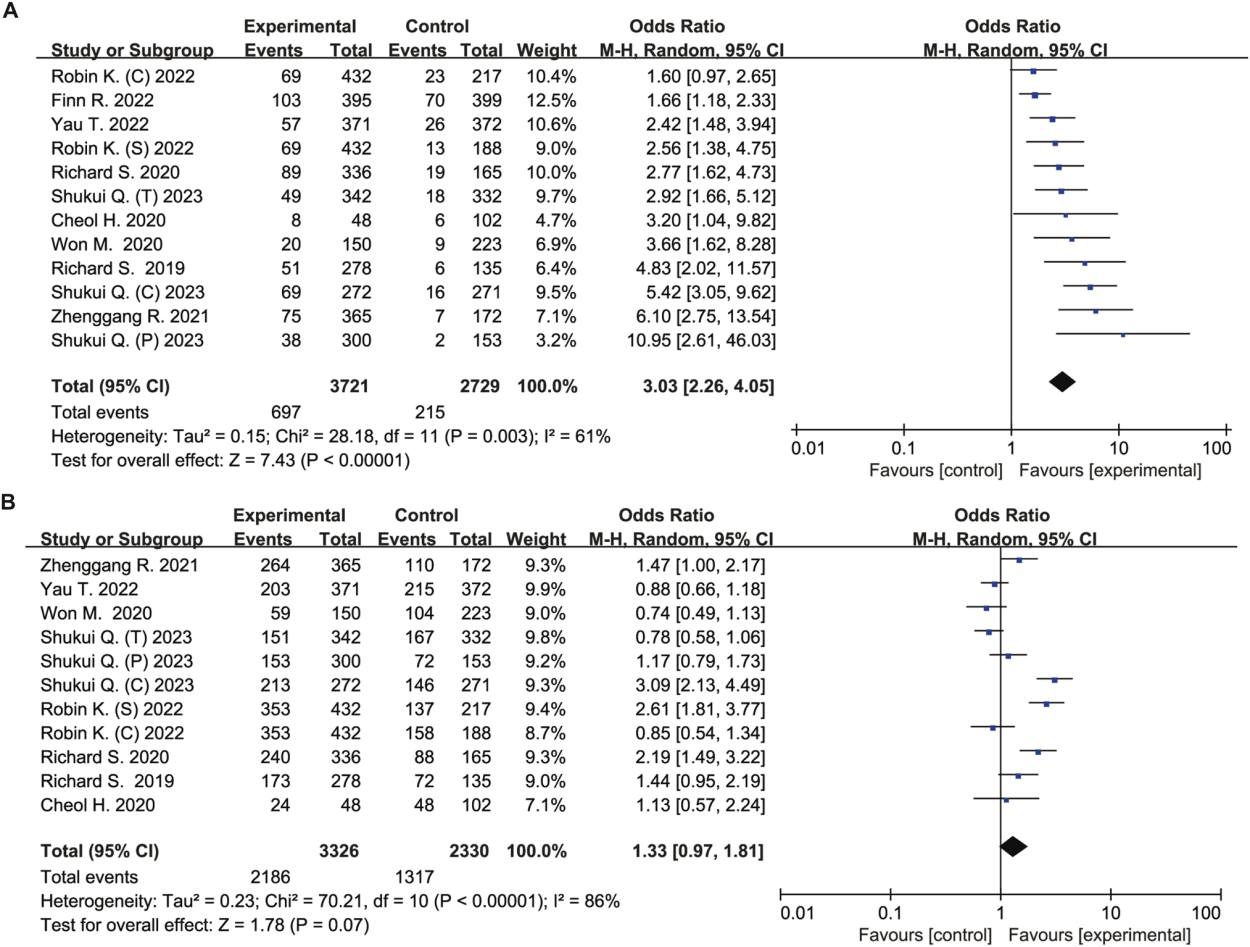
Forest plot of meta-analysis for ORR and DCR. **A** Objective response rate. **B** Disease control rate

There was a significant difference between the intervention group and control group in terms of ORR (OR 3.03, 95% CI 2.26–4.05, *P* < 0.00001), but DCR (OR 1.33, 95% CI 0.97–1.81, *P* = 0.07). Significant heterogeneities were observed in ORR (I^2^ = 61%) and DCR (I^2^ = 86%) (Fig. 2).

### SD and PD

**Fig. 3:**
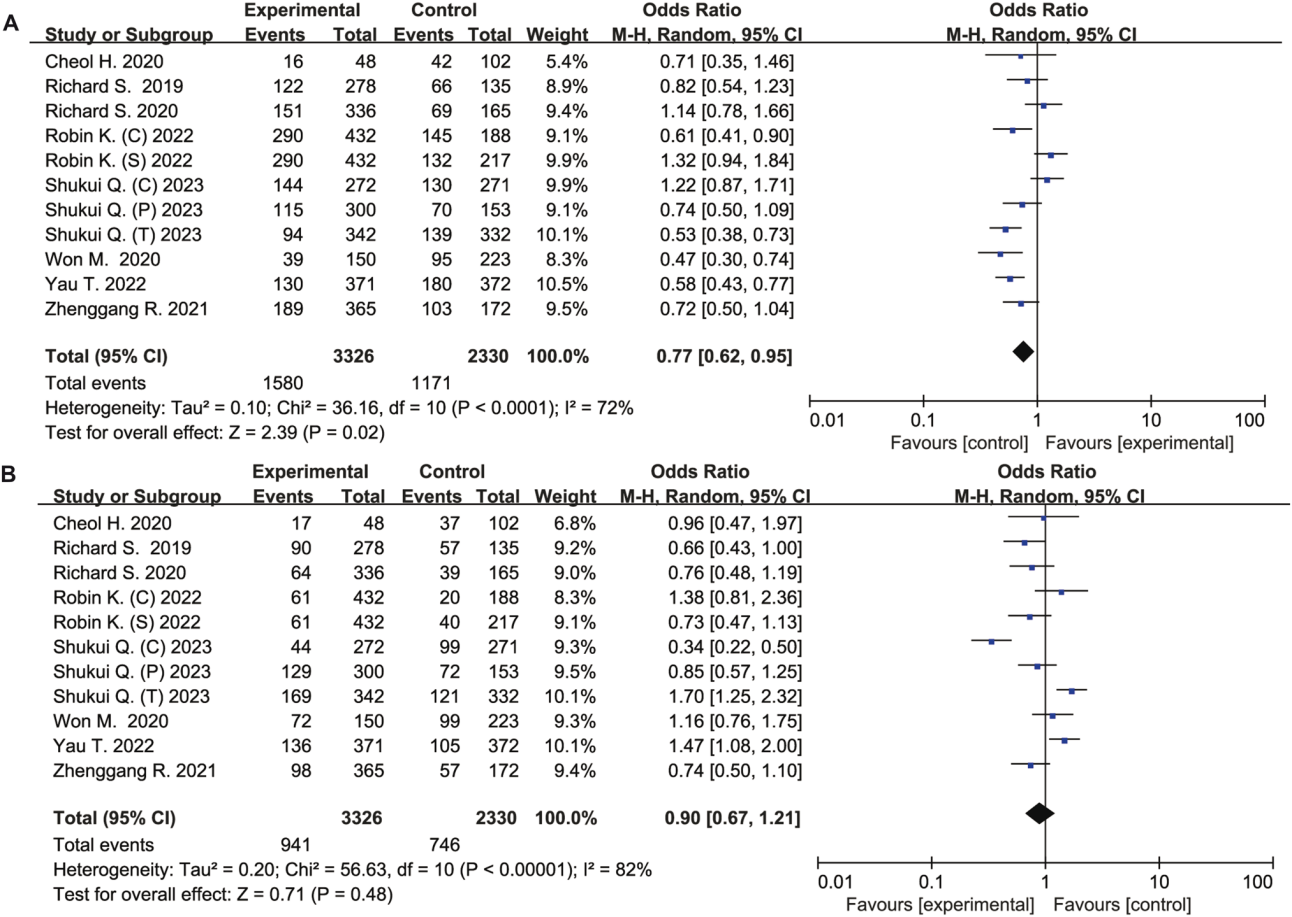
Forest plot of meta-analysis for SD and PD. **A** Stable disease. **B** Progressive disease

HCC patients treated with immune checkpoint inhibitors had significantly different levels of SD (OR 0.77, 95% CI 0.62–0.95, *P* = 0.02), but PD (OR 0.90, 95% CI 0.67–1.21, *P* = 0.48) (Fig. 3).

### Adverse events

**Fig. 4:**
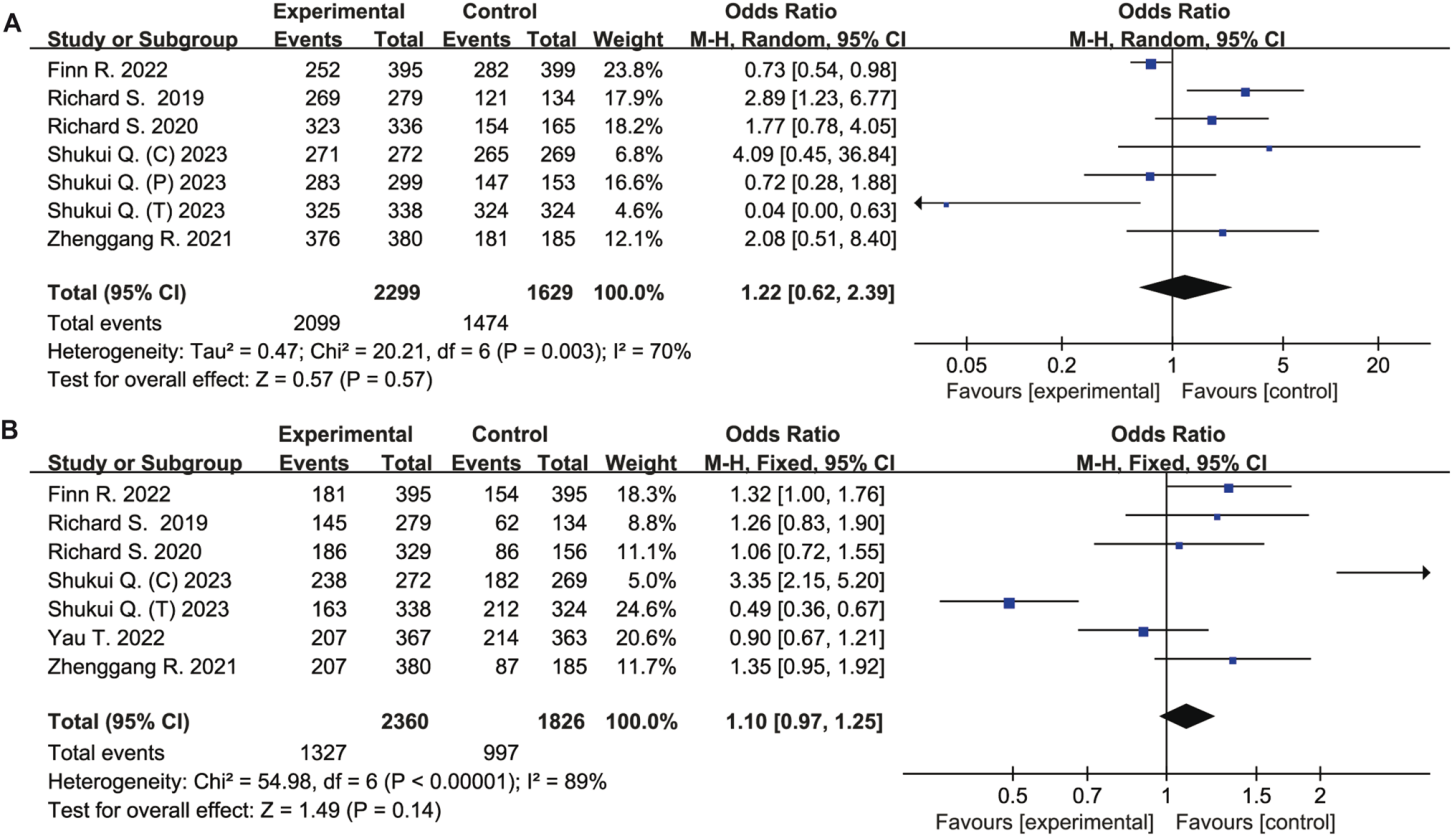
Forest plot of meta-analysis for any caused adverse events. **A** Any-grade adverse events. **B** All caused ≥ grade 3 adverse events

**Fig. 5:**
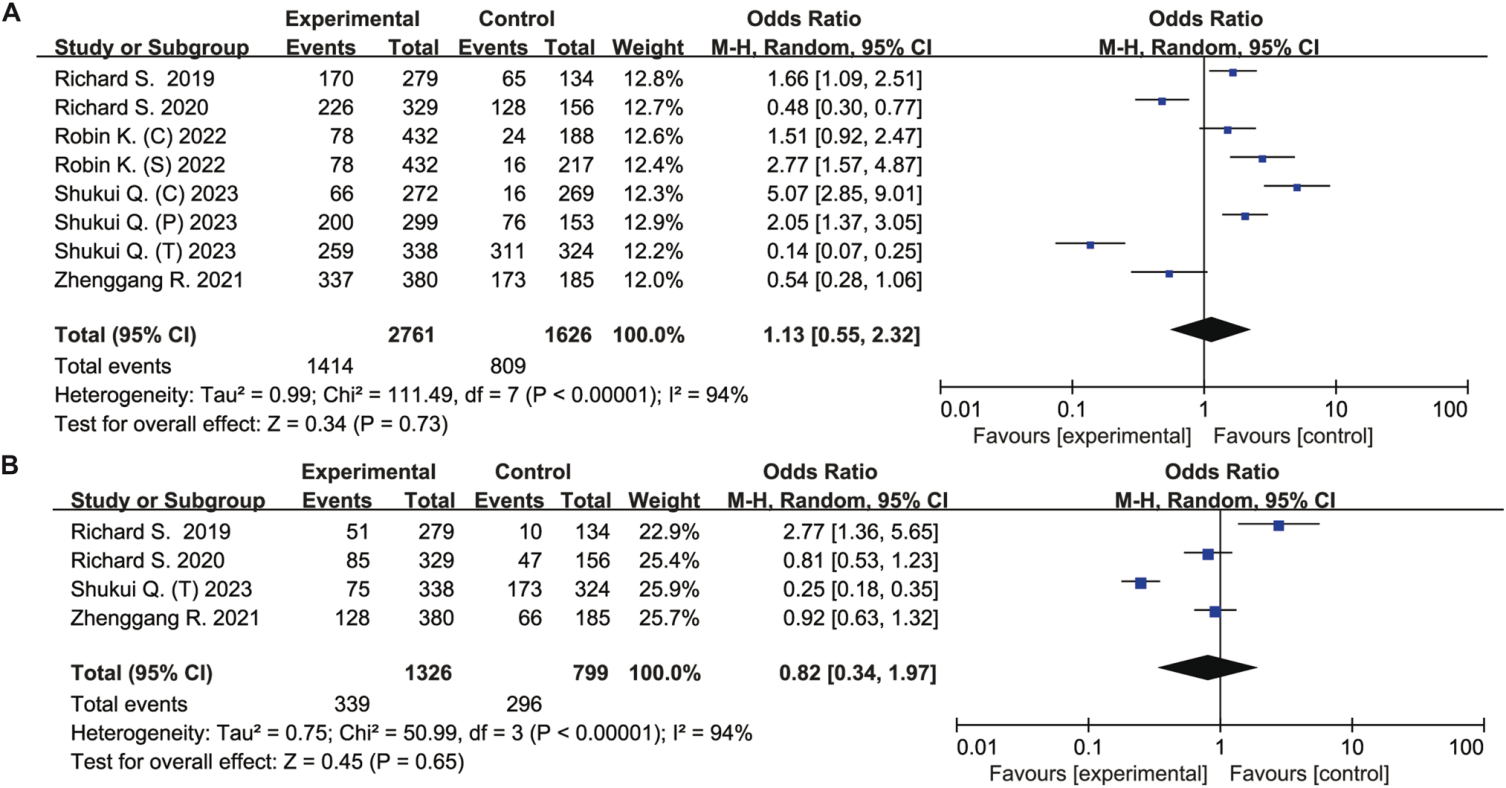
Forest plot of meta-analysis for treatment-related adverse events. **A** Any-grade adverse events. **B** Treatment-related ≥ grade 3 events

Compared to the control group, the ICIs group did not increased risk of all caused any-grade adverse events (OR 1.22, 95% CI 0.62–2.39, *P* = 0. 57), all caused ≥ grade 3 adverse events (OR 1.10, 95% CI 0.97–1.25, *P* = 0.14), treatment-related any-grade adverse events (OR 1.13, 95% CI 0.55–2.32, *P* = 0.73), and treatment-related ≥ grade 3 events (OR 0.82, 95% CI 0.34–1.97, *P* = 0.65) (Figs. 4, 5).

### OS and PFS

**Fig. 6:**
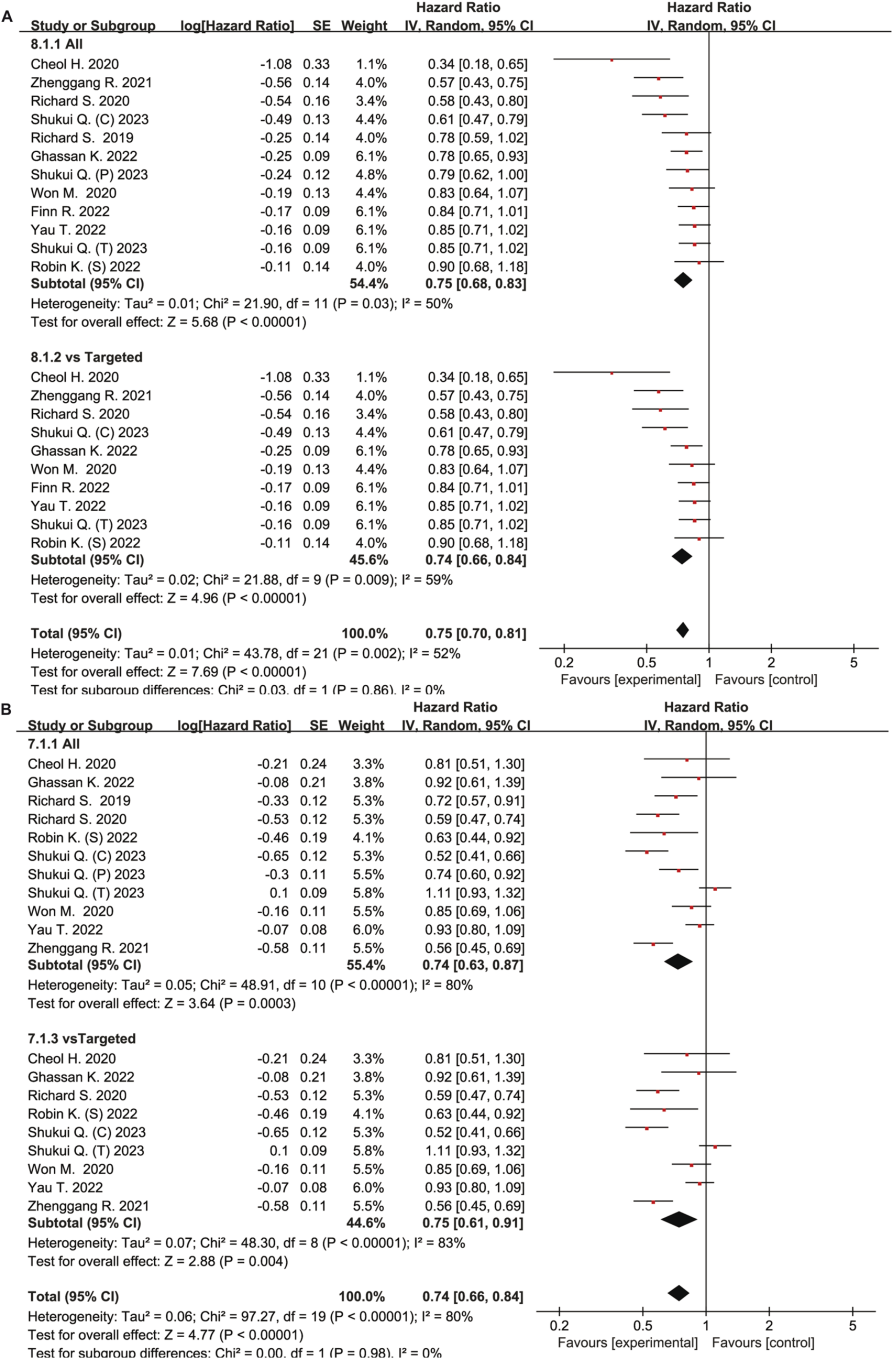
Forest plot of meta-analysis for OS and PFS. **A** Over survival (OS). **B** Progression-free survival

The effect size was calculated as HRs. There was no significant difference between intervention group and control group OS (HR 0.75, 95% CI 0.68–0.83, P < 0.00001) and PFS (HR 0.74, 95% CI 0.63–0.87, *P* < 0.0003). However, after the removal of un-targeted drug in control group, the subgroup analysis revealed that the intervention group had longer OS (HR 0.74, 95% CI 0.66–0.84, *P* < 0.00001) and PFS (HR 0.75, 95% CI 0.61–0.91, *P* = 0.004). (Fig. 6). Additionally, a subgroup analysis of population characteristics was carried out (Figures S2–9). The subgroup analysis determined that the gender, patients with an ECOG = 0 or ECOG score >  = 1, HBV-positive patients, and microvascular-invasion positive or negative HCC patients in the intervention group had a longer PFS, whereas no significant differences were noted in HBV-negative patients.

### Assessment of study quality, publication bias, and sensitivity

The quality assessment of 12 studies is summarized in Table S3, while the risk of bias is summarized in Figure S1. The pooled analysis of DCR, SD, PD, OS, any-grade adverse events, all caused ≥ grade 3 adverse events, treatment-related any-grade adverse events, and PFS did not show evidence of publication bias based on the funnel plot and Egger’s tests. However, there was significant publication bias observed in the meta-analysis of ORR and OS (Figure S10–19). A sensitivity analysis was performed, which showed that ORR, SD, OS, and the subgroup of PFS were robust to the decisions made during the process. However, the results for ORR, PD, any-grade adverse events, all caused ≥ grade 3 adverse events, treatment-related any-grade adverse events, treatment-related ≥ grade 3 events, and PFS from all included studies should be interpreted with caution due to potential bias.

## Discussion

Hepatocellular carcinoma (HCC) is a growing global health concern, with its incidence rates experiencing an estimated annual increase of 2% to 3% based on data from the national center for health statistics of the US (Liao et al. 2021; Wang et al. 2021). Despite a slight slowdown in recent years, this upward trend remains concerning. The development of hepatic tumors not only impacts the patient's digestive and immune systems but also causes significant pain (Cheng et al. 2021). Consequently, the search for effective treatments for liver cancer has been a longstanding objective for medical practitioners and scientists. Thankfully, the emergence of immune checkpoint inhibitors has brought about a paradigm shift in cancer treatment.

The current primary targets of immune checkpoint inhibitors are PD-1 (programmed death protein 1), PD-L1 (programmed death ligand 1), and CTLA-4 (cytotoxic T-lymphocyte antigen 4), which are proteins predominantly expressed by the immune system (Skafi et al. 2020). These proteins regulate and restrain an overly aggressive immune response that could mistakenly attack healthy cells within the body. The process of immune checkpoint signaling is initiated when proteins on the surface of T cells recognize and bind to complementary proteins on other cells, including cancer cells. This binding event triggers a signal cascade that suppresses T cell activity, dampening the immune response and safeguarding the target cell from attack (Lee et al. 2019). Leveraging this characteristic of T cells, scientists have developed inhibitors designed to block these immune checkpoints, commonly known as immune checkpoint inhibitors. Notably, several immune checkpoint inhibitors, such as nivolumab (PD-1), ipilimumab (CTLA-4), pembrolizumab (PD-1), atezolizumab (PD-L1), sintilimab (PD-1), and camrelizumab (PD-1), have obtained approval in various countries and regions for the treatment of HCC.

In our research, we conducted a comprehensive investigation of ORR, DCR, SD, PD, PFS, OS, and adverse events in 6,018 hepatocellular carcinoma (HCC) patients across 12 studies (Abou-Alfa et al. 2022; Choi et al. 2020; Finn et al. 2022, 2020a, 2020b; Kelley et al. 2022; Lee et al. 2020; Qin et al. 2023a, 2023b, 2023c; Ren et al. 2023; Yau et al. 2022).

ORR, a well-established metric for evaluating tumor burden in patients with solid tumors following specific treatments, initially posed challenges for immune checkpoint inhibitors due to lower patient response rates compared to targeted drugs. Meta-analyses have indicated a statistically significant difference in ORR (P < 0.00001), demonstrating a higher ORR in patients receiving immune checkpoint inhibitors compared to those treated with targeted drugs, thus supporting the favorable response of HCC patients to immune checkpoint inhibitors.

DCR, a measure assessing the proportion of patients achieving a partial response, complete response, or stable disease, has yielded conflicting findings in the literature. In our study, we found no significant differences in PD and DCR between the group receiving immune checkpoint inhibitors and the control group. However, we observed that the SD in the control group was superior to that in the immune checkpoint inhibitors group.

Specific drugs examined in our study demonstrated varying DCR rates. Cabozantinib + atezolizumab exhibited the highest DCR at 82%, while sorafenib and cabozantinib achieved DCR rates of 63% and 84%, respectively (Kelley et al. 2022). Additionally, regorafenib and nivolumab demonstrated the highest rates of SD at 42.6% and 26%, respectively (Choi et al. 2020). Notably, nivolumab had the highest PD rate at 37%, while sorafenib had a PD rate of 28% (Yau et al. 2022). It is important to consider the potential influence of pseudoprogression on the assessment of DCR, SD, and PD in some patients. Pseudoprogression, though rare in cancer patients treated with immune checkpoint inhibitors, should be distinguished from actual tumor progression, as it has been reported in approximately 10% of cases in previous studies (Frelaut et al. 2020).

The assessment of DCR, SD, and PD based on Response Evaluation Criteria in Solid Tumors (RECIST) may introduce bias when applied to the evaluation of immune checkpoint inhibitors due to their distinct mechanisms compared to traditional drugs. Therefore, there is an urgent need to establish evaluation indicators suitable for assessing the therapeutic effects of immune checkpoint inhibitors. While various clinical efficacy evaluation criteria for immunotherapy, such as immune-related response criteria (irRC criteria), immune-related RECIST (irRECIST), immune-modified RECIST (iRECIST), and immune-modified RECIST (imRECIST) for solid tumors, are available, the studies included in our analysis relied on the RECIST criteria.

In the analysis of patient outcomes, notable divergences in both progression-free survival (PFS) and overall survival (OS) were observed. This finding is substantiated by accordance to prior research, including studies by He et al. (2021), Jácome et al. (2021), and Rao et al. (2020). However, the considerable heterogeneity, as indicated by an I2 value exceeding 50% among the reviewed studies, necessitated a further investigation of subgroups. Notably, the majority of control groups employed targeted drugs or placebos, due to the regulatory approval and established efficacy of targeted drugs prior to the introduction of immune checkpoint inhibitors (ICIs) in the treatment of hepatocellular carcinoma (HCC).

Subsequently, a subgroup analysis was conducted to evaluate the potential superiority of ICIs compared to targeted drugs in the treatment of HCC patients, revealing an initial advantage of ICIs over targeted drugs, albeit with a tendency to diminish over time. This phenomenon aligns with expectations, given the well-established efficacy of targeted drugs as primary therapies for HCC prior to the advent of ICIs. Indeed, the confluence of clinical trials and retrospective analyses underscores the indispensable role of targeted drugs in the treatment of HCC.

Furthermore, emerging evidence suggests that combining ICIs with drugs targeting vascular endothelial growth factor (VEGF) or epidermal growth factor receptor (EGFR) may yield significantly enhanced clinical benefits (Donne and Lujambio 2023; Tran et al. 2022; Yu 2023). However, the present study did not encompass an investigation into the combination of ICIs with VEGF-targeting drugs due to the limited scope of the inclusion criteria. Subsequently, a subgroup analysis focusing on patient characteristics was performed to assess PFS, revealing correlations between PFS and gender, Eastern Cooperative Oncology Group (ECOG) score, history of hepatitis B virus (HBV) infection, and the presence or absence of macrovascular invasion in HCC. Notably, HBV-negative status did not demonstrate a correlation with PFS, prompting the integration of quantitative assessments of physical parameters in the drug selection process for HCC patients prior to the administration of ICIs.

The safety profile of the interventions was evaluated through an assessment of all-grade adverse events and treatment-related adverse events. The results indicated no substantial differences between the ICIs group and the control group in terms of all-grade adverse events, all-grade adverse events of grade 3 or higher, treatment-related adverse events of any grade, and treatment-related adverse events of grade 3 or higher. Despite these manageable side effects, it is imperative for the medical team to remain attentive to certain distinct adverse reactions associated with immune checkpoint inhibitors, such as diarrhea, as well as aspartate aminotransferase (AST) and alanine aminotransferase (ALT) elevation. Formulating an effective treatment strategy and ensuring adept clinical care necessitates heightened vigilance toward these adverse reactions.

However, it is important to acknowledge the limitations of our study. Firstly, liver cancer is a complex tumor with various functions performed by the liver in the body. Unfortunately, due to limitations in the reported results of the eligible studies, factors such as ethnicity, viral infection, and tumor stage that can influence treatment outcomes were only partially included in this study. Existing studies have shown that different drugs have different therapeutic effects depending on these factors, highlighting the need for further research in this area to achieve precise therapeutic goals.

Secondly, there was high heterogeneity observed, as demonstrated in the forest plot of the results section. Subgroup analyses were conducted to address this issue, but the heterogeneity could only be partially reduced. This may introduce some degree of uncertainty in the findings and should be taken into consideration in the interpretation of the results.

Thirdly, the primary objective of this study was to investigate the therapeutic effect of immune checkpoint inhibitors (ICIs) on hepatocellular carcinoma (HCC). However, due to the limited screening conditions, the drug ipilimumab was not included in the analysis. We acknowledge this limitation and plan to reanalyze the data in the future to obtain higher-grade evidence and a more comprehensive understanding of the therapeutic effects of ICIs on HCC.

Despite these limitations, our study followed relatively rigorous inclusion criteria, resulting in a small number of included studies but with a higher quality of evidence. By combining the findings of our study with published meta-analyses of ICIs for HCC, we still believe that ICIs can provide benefits in the treatment of HCC, even though this was not the primary endpoint of our study. However, when compared to other types of cancer such as lung cancer and breast cancer, HCC still has suboptimal overall survival rates and survival times. Therefore, further studies are warranted to improve our understanding and develop more effective treatment strategies for HCC.

### Supplementary Information

Below is the link to the electronic supplementary material.

Supplementery file1. Table S1**.** Search terms. **Table S2.** Published meta-analysis of ICIs treatment of HCC. **Table S3.** GRADE evidence assessment. Figure S1. The risk of bias. **Figure S2.** subgroup analysis of ECOG = 0 in PFS. **Figure S3.** subgroup analysis of ECOG ≥ 1 in PFS. **Figure S4,** subgroup analysis of male in PFS. **Figure S5.** subgroup analysis of female in PFS. **Figure S6**, subgroup analysis of HBV positive in PFS. **Figure S7.** subgroup analysis of HBV negative in PFS. **Figure S8**, subgroup analysis of macrovascular invasion positive in PFS. **Figure S9**. subgroup analysis of macrovascular invasion negative in PFS. Figure S1**0**. publication bias and sensitivity analysis of ORR (A. Publication bias, *P* = 0.0042. B. Sensitivity). Figure S1**1**. Publication bias and sensitivity analysis of DCR (A. Publication bias,* P* = 0.6625. B. Sensitivity). Figure S1**2.** Publication bias and sensitivity analysis of SD (A. Publication bias,* P* = 0.8670. B. Sensitivity). Figure S1**3.** Publication bias and sensitivity analysis of PD (A. Publication bias,* P* = 0.2652. B. Sensitivity). Figure S1**4**. publication bias and sensitivity analysis of any-grade adverse events (A. Publication bias,* P* = 0.4690. B. Sensitivity). Figure S1**5**. publication bias and sensitivity analysis of all caused ≥ grade 3 adverse events (A. Publication bias,* P* = 0.2126. B. Sensitivity). Figure S1**6.** publication bias and sensitivity analysis of treatment-related any-grade adverse events (A. Publication bias,* P* = 0.4524. B. Sensitivity). Figure S1**7**. sensitivity analysis of treatment-related ≥ grade 3 events. Figure S18, sensitivity analysis of OS (A. Publication bias,* P* = 0.0058. B. Sensitivity of all studies. C. Sensitivity of subgroup). Figure S1**9.** sensitivity analysis of PFS (A. Publication bias,* P* = 0.3179. B. Sensitivity of all studies. C. Sensitivity of subgroup).

Supplementary file2 (DOCX 31.6 KB)

## Data Availability

All authors had access to the data published in this paper.
