# Supplementary material for: Approved immune checkpoint inhibitors in hepatocellular carcinoma: a large-scale meta-analysis and systematic review
Source: J Cancer Res Clin Oncol. 2024 Feb 6;150(2):82. doi: 10.1007/s00432-023-05539-8 (PMC10847200; doi:10.1007/s00432-023-05539-8)

**Supplemental table 1. search terms.**

| **PubMed:**  (hepatocellular carcinoma OR "Carcinoma, Hepatocellular"[MeSH] OR "Liver Neoplasms"[MeSH]) AND (Nivolumab OR Durvalumab OR Tislelizumab OR Ipilimumab OR Pembrolizumab OR Atezolizumab OR Camrelizumab OR Tremelimumab OR Sintilimab) NOT ((animals [mh] NOT humans [mh])) NOT (systematic[sb] OR Editorial[pt] OR Meta-Analysis[pt] OR Review[pt] OR Case Reports[pt] OR Letter[pt] OR Systematic Review [pt]) |
| --- |
| **Embase:**  **#1**  ('hepatocellular carcinoma'/exp OR 'hepatocellular carcinoma' OR (hepatocellular AND ('carcinoma'/exp OR carcinoma))) NOT ([animals]/lim NOT [humans]/lim) NOT ([editorial]/lim OR [erratum]/lim OR [letter]/lim OR [review]/lim OR [short survey]/lim)  **#2**  #1 AND 'nivolumab'/exp OR nivolumab OR 'durvalumab'/exp OR durvalumab OR 'tislelizumab'/exp OR tislelizumab OR 'ipilimumab'/exp OR ipilimumab OR 'pembrolizumab'/exp OR pembrolizumab OR 'atezolizumab'/exp OR atezolizumab OR 'camrelizumab'/exp OR camrelizumab OR 'tremelimumab'/exp OR tremelimumab OR 'sintilimab'/exp OR sintilimab |
| **Cochrane:**  **#1**  "Liver Cell Carcinoma" OR "Hepatocellular Carcinomas" OR "Liver Cancer"  **#2**  (Nivolumab OR ONO 4538 OR MDX 1106 OR BMS936558 OR Ipilimumab OR Yervoy OR MDX 010 OR MDX CTLA 4 OR pembrolizumab OR SCH-900475 OR lambrolizumab OR MK-3475 OR Keytruda OR atezolizumab OR MPDL3280A OR RG7446 OR Camrelizumab OR SHR 1210 OR Tremelimumab OR CP 675 OR CP 675206 OR sintilimab OR IBI 308 OR durvalumab OR Imfinzi OR MEDI4736 OR Tislelizumab OR BGB-A317)  **#3**  #1 AND #2 |

**Supplemental table 2. Published meta-analysis of ICIs treatment of HCC**

| **(Serial number)**  **Author** | **Year** | **NO. of studies** | **Indicator** | **Experimental** | **Control** | **Conclusion** | **Notes** |
| --- | --- | --- | --- | --- | --- | --- | --- |
| (1) Feng Z. | 2019 | 13 | ORR, DCR, OS, PFS, TrAEs | ICIs | ICIs+ anti-  VEGF agents | (1) Combination of PD-1/PD- L1 inhibitors with anti-VEGF agents results in clinically significant improvements in certain outcomes in HCC. (2) The risk of immune related toxicity was increased. |  |
| (2) Robin P. | 2021 | 13 | ORR, OS, PFS | Systemic agents | Placebo | Atezolizumab plus bevacizumab appears to have superior efficacy among first line agents whereas cabozantinib appears to be superior in the second line setting | Network  Meta-Analysis |
| (3) Quan R. | 2020 | 20 | CR, PR, SD, ORR, DCR, OS, PFS, AE, SAE | PD‑1/PD‑L1 inhibitors | NA | PD‑1/PD‑L1 inhibitors showed favorable outcomes concerning response rates and survival periods in advanced HCC. | Most are single-arm trials |
| (4) Hang Y. | 2020 | 8 | AE, FAES | ICIs |  | (1) ICIs signiﬁcantly increases the risk of AEs in advanced HCC patients. (2) The risk of grade ≥ 3 AEs is associated with Child—Pugh classiﬁcation | Advanced hepatocellular carcinoma |
| (5) Ziniu D. | 2021 | 8 | ORR | Virus positive | Virus Negative | Viral etiology may not be considered as the selection criteria for patients receiving ICIs in HCC | Most are early-stage clinical trials |
| (6) Ioannis A. | 2019 | 3 | RR, DCR, OS, PFS | Pembrolizumab/Nivolumab | Sorafenib | ICIs did not show an advantage for HCC | Pembrolizumab and nivolumab are the only ICIs involved in this study |
| (7) AlexandreA. | 2021 | 3 | OS, PFS, ORR, TRAEs | ICIs | Sorafenib/ Placebo | (1) ICIs has shown superior safety and efficacy compared to standard therapy in HCC. (2) highlights the survival benefit associated with the combination of antiangiogenic therapy with ICIs infirstline systemic therapy of unresectable HCC | Pembrolizumab, nivolumab or Atezolizumab + bevacizumab involved in this study |
| (8) Shukang H. | 2021 | 12 | RR, DCR, OS, PFS, TRAEs | ICIs | Targeted/ Placebo | (1) HCC patients would beneﬁt from ICIs treatment | 8 were single-arm studies, 2 were RCTs and 2 were retrospective cohort studies |
| (9) Jiaxi Z. | 2022 | 29 | ORR, DCR, OS, PFS | ICIs | Placebo/ Single arm/Targeted | ICIs combination therapy improved clinical outcomes in advanced HCC. | Control group diversification |
| (10) Won J. | 2020 | 6 | ORR | Virus positive | Virus Negative | Viral status should not be used as a criterion to select patients for PD-1/PD-L1 therapy | Meta-analysis is only part of the study |
| (11) BaoWen T. | 2023 | 41 | OS, PFS | ICIs/ Combination | NA | Although the survival time of patients with impaired liver function may be relatively short, ICIs still have great potential for therapeutic applications. | (1) All included studies were retrospective. (2) The main concern is the effect of liver function on the therapeutic effect |
| (12) Wen W. | 2023 | 13 | ORR, DCR, OS, PFS, ImAE | PD-1/PD-L1/ Combination | Different dose ICIs/ Placebo | PD-L1/PD-1 inhibitors were effective and tolerable in patients with advanced HCC | Most are Phase Ⅰ or Phase Ⅱ trials |
| (13) Linyan Z. | 2022 | 3 | ORR, DCR, OS, PFS, AE, SAE | Atezolizumab+bevacizumab/ Pembrolizumab/ Nivoluma | Sorafenib/ Placebo | (1) PD-L1 inhibitor combined with anti-VEGF antibody could improve the prognosis of patients with uHCC. (2) Caution should be taken for AEs during patients receiving PD-1/PD-L1 inhibitors. | Only 3 studies were involved |
| (14) Yuwei L. | 2023 | 98 | ORR, DCR, OS, PFS, AE, SAE | PD-1/PD-L1/ Combination | Different dose ICIs/ Placebo/ Targeted | PD-1/PD-L1 inhibitors should be the preferred treatment choice for advanced HCC owing to their higher antitumor effect and improved outcomes | Too many low-quality studies were included |
| (15) Danxue H. | 2023 | 5 | ORR, DCR, OS, PFS, TRAEs | PD-1/PD-L1/Combination | Sorafenib | PD-1/PD-L1 inhibitors combination therapy for unresectable HCC was associated with better clinical outcomes than anti-angiogenic monotherapy, especially for HBV infection and Asian population. | All the studies were phase III randomized clinical trials |
| (16) Y-H H. | 2023 | 4 | MPR, pCR, ORR, TRAE | ICI/Combination | NA | This meta-analysis provides preliminary evidence of the efficacy and safety of neoadjuvant immunotherapy for HCC, suggesting that it is a promising perioperative treatment option | Focus on resectable HCC. Early clinical trials to study dose safety and efficacy |
| (17) Xiaoqiang G. | 2023 | 23 | ORR, CR, PR, OS, PFS, AE, SAE | Atezolizumab+bevacizumab | NA | Atezolizumab in combination with bevacizumab showed good efficacy and tolerability in the treatment of advanced HCC | Most are single-arm studies |
| (18) Sihao D. | 2023 | 8 | ORR, DCR, OS, PFS, AE, SAE | Atezolizumab+bevacizumab | lenvatinib | Our study did not find any significant difference in effectiveness and safety between Atez/Bev and lenvatinib | All the studied were retrospectively |
| (19) Alessandro R. | 2023 | 3 | Grade 3/4 TRAEs, grade 5 TRAEs, serious TRAEs, TRAEs leading to discontinuation | ICI/Combination | Sorafenib | Beyond activity and efficacy, careful consideration should be given to toxicity while choosing the appropriate first-line treatment in HCC | Focus on TRAEs. |

AE: adverse events. SAE: serious adverse events. FAEs: fatal adverse events. CR: complete response. PR: partial response. RR: response rate. ImAE: Immune‑mediated adverse events. TRAEs: treatment-related adverse events. DFS: disease-free survival. MPR: major pathological response. pCR: pathological complete response.

(1) Meta-analysis of the efficacy and safety of PD-1/PD-L1 inhibitors administered alone or in combination with anti-VEGF agents in advanced hepatocellular carcinoma. doi: 10.1136/gutjnl-2019-320116.

(2) Comparison of Efficacy of Systemic Therapies in Advanced Hepatocellular Carcinoma: Updated Systematic Review and Frequentist Network Meta-Analysis of Randomized Controlled Trials. doi: 10.2147/JHC.S268305. eCollection 2021.

(3) Clinical benefits of PD‑1/PD‑L1 inhibitors in advanced hepatocellular carcinoma: a systematic review and meta‑analysis. doi: 10.1007/s12072-020-10064-8.

(4) Risk of adverse events in advanced hepatocellular carcinoma with immune checkpoint therapy: A systematic review and meta-analysis. doi: 10.1016/j.clinre.2020.02.012. Epub 2020 Apr 16.

(5) Viral Status and Efﬁcacy of Immunotherapy in Hepatocellular Carcinoma: A Systematic Review With Meta-Analysis. doi: 10.3389/fimmu.2021.733530.

(6) PD-1 inhibitors monotherapy in hepatocellular carcinoma: Meta-analysis and systematic review. doi: 10.1016/j.hbpd.2019.09.007. Epub 2019 Sep 14.

(7) Efficacy and Safety Associated With Immune Checkpoint Inhibitors in Unresectable Hepatocellular Carcinoma A Meta-analysis. doi: 10.1001/jamanetworkopen.2021.36128.

(8) The Efﬁcacy and Safety of Programmed Death-1 and Programmed Death Ligand 1 Inhibitors for the Treatment of Hepatocellular Carcinoma: A Systematic Review and Meta Analysis. doi: 10.3389/fonc.2021.626984. eCollection 2021.

(9) Benefits of combination therapy with immune checkpoint inhibitors and predictive role of tumour mutation burden in hepatocellular carcinoma: A systematic review and meta-analysis. doi: 10.1016/j.intimp.2022.109244.

(10) Viral status, immune microenvironment and immunological response to checkpoint inhibitors in hepatocellular carcinoma. doi: 10.1136/jitc-2019-000394.

(11) Evaluating liver function and the impact of immune checkpoint inhibitors in the prognosis of hepatocellular carcinoma patients: A systemic review and meta-analysis. doi: 10.1016/j.intimp.2022.109519.

(12) Clinical outcomes of PD-1/PD-L1 inhibitors in patients with advanced hepatocellular carcinoma: a systematic review and meta-analysis. doi: 10.1007/s00432-022-04057-3. Epub 2022 Jun 30.

(13) Survival Outcomes and Safety of Programmed Cell Death/Programmed Cell Death Ligand 1 Inhibitors for Unresectable Hepatocellular Carcinoma: Result From Phase III Trials. doi: 10.1177/10732748221092924.

(14) Efficacy and Safety of PD-1/PD-L1 Inhibitors in Advanced Hepatocellular Carcinoma: A Systematic Review and Meta-analysis. doi: 10.1007/s12325-022-02371-3.

(15) Efficacy and safety of PD-1/PD-L1 inhibitors combined with anti-angiogenic therapy for the unresectable hepatocellular carcinoma and the benefit for hepatitis B virus etiology subgroup: a systematic review and meta-analysis of randomized controlled trials. doi: 10.1186/s12885-023-10960-w.

(16) Neoadjuvant immunotherapy for resectable hepatocellular carcinoma: a systematic review and meta-analysis. doi: 10.26355/eurrev_202308_33287.

(17) Efficacy and safety of atezolizumab plus bevacizumab treatment for advanced hepatocellular carcinoma in the real world: a single-arm meta-analysis. doi: 10.1186/s12885-023-11112-w.

(18) Clinical efficacy and safety of atezolizumab plus bevacizumab versus lenvatinib in the treatment of advanced hepatocellular carcinoma: A systematic review and meta-analysis. doi: 10.1097/MD.0000000000033852.

(19) Treatment-related adverse events of first-line immunotherapy versus sorafenib for advanced hepatocellular carcinoma: a meta-analysis. doi: 10.1080/14740338.2023.2152793. Epub 2022 Nov 29.

**Supplemental table 3. GRADE evidence assessment.**

| **Outcome** | **NO. of studies** | **Study design** | **Risk of bias** | **Inconsistency** | **Indirectness** | **Imprecision** | **Other considerations** | **Odds ratio (95% CI)** | **Certainty** | **Importance** |
| --- | --- | --- | --- | --- | --- | --- | --- | --- | --- | --- |
| ORR | 10 | RCT | serious | not serious | not serious | not serious | none | 3.00  [2.16, 4.16] | ⨁⨁⨁◯  Moderate | IMPORTANT |
| ORR | 2 | observational studies | not serious | serious | serious | not serious | none | 6.42  [3.06 -13.48] | ⨁◯◯◯  Very low | NOT IMPORTANT |
| DCR | 9 | RCT | serious | not serious | not serious | not serious | none | 1.43  [1.02, 2.02] | ⨁⨁⨁◯  Moderate | IMPORTANT |
| DCR | 2 | observational studies | not serious | serious | serious | not serious | none | 0.83  [0.58 - 1.19] | ⨁◯◯◯  Very low | NOT IMPORTANT |
| SD | 9 | RCT | serious | not serious | not serious | not serious | none | 0.81  [0.64, 1.02] | ⨁⨁◯◯  Low | NOT IMPORTANT |
| SD | 2 | observational studies | not serious | no serious | serious | not serious | none | 0.53  [0.36 - 0.78] | ⨁◯◯◯  Very low | NOT IMPORTANT |
| PD | 9 | RCT | serious | not serious | not serious | serious | none | 0.87  [0.61, 1.23] | ⨁⨁◯◯  Low | NOT IMPORTANT |
| PD | 2 | observational studies | not serious | no serious | serious | not serious | none | 1.10  [0.77 -1.58] | ⨁◯◯◯  Very low | NOT IMPORTANT |
| Adverse events | 7 | RCT | not serious | not serious | not serious | not serious | none | 1.22  [0.62, 2.39] | ⨁⨁◯◯  Low | IMPORTANT |
| Related adverse events | 8 | RCT | serious | serious | not serious | serious | none | 1.13  [0.55, 2.32] | ⨁⨁◯◯  Low | NOT IMPORTANT |
| OS | 10 | RCT | serious | serious | not serious | serious | none | 0.77  [0.70, 0.84] | ⨁⨁⨁◯  Moderate | IMPORTANT |
| OS | 2 | observational studies | serious | not serious | serious | not serious | none | 1.78  [0.74 - 4.29] | ⨁◯◯◯  Very low | NOT IMPORTANT |
| PFS | 11 | RCT | serious | not serious | not serious | not serious | none | 0.73  [0.60, 0.88] | ⨁⨁⨁◯  Moderate | IMPORTANT |
| PFS | 2 | observational studies | serious | not serious | serious | not serious | none | 1.59  [0.84 - 3.00] | ⨁◯◯◯  Very low | NOT IMPORTANT |

**Supplemental figure 1, the risk of bias.**


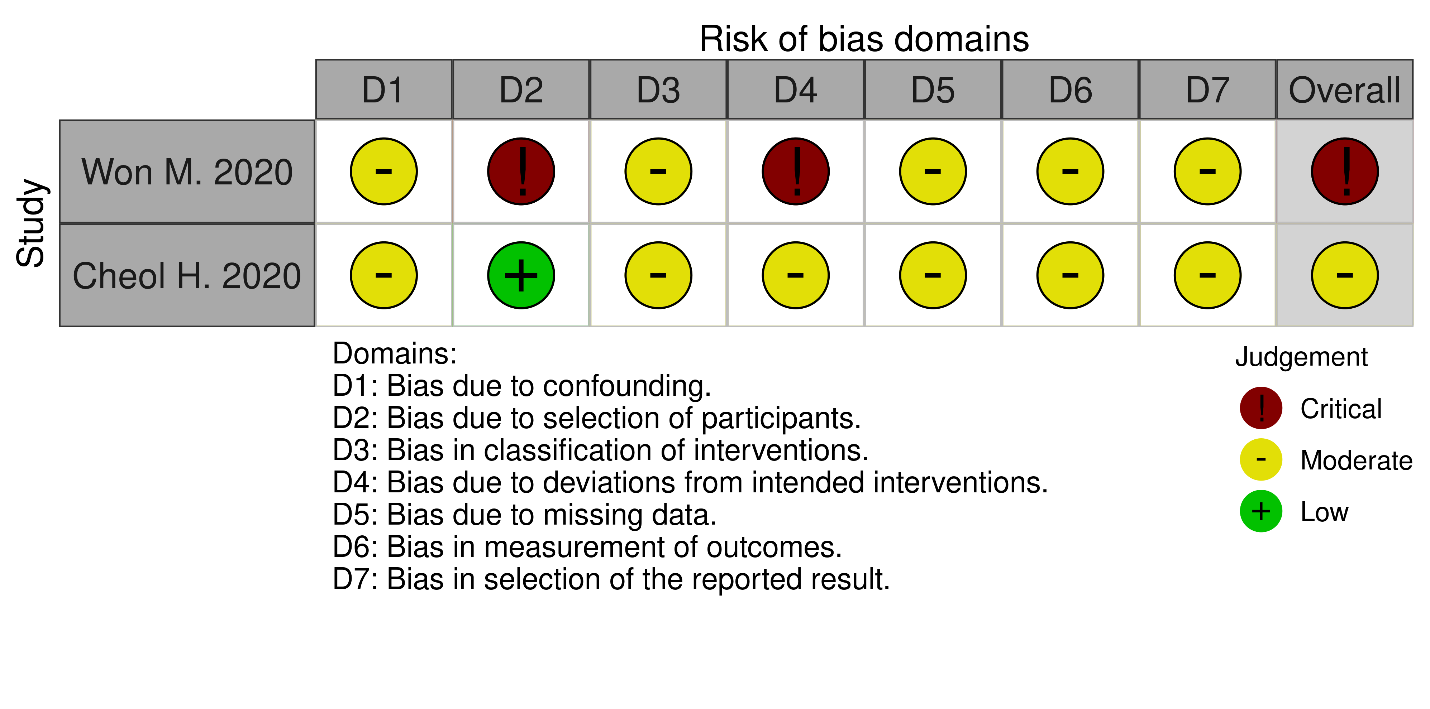


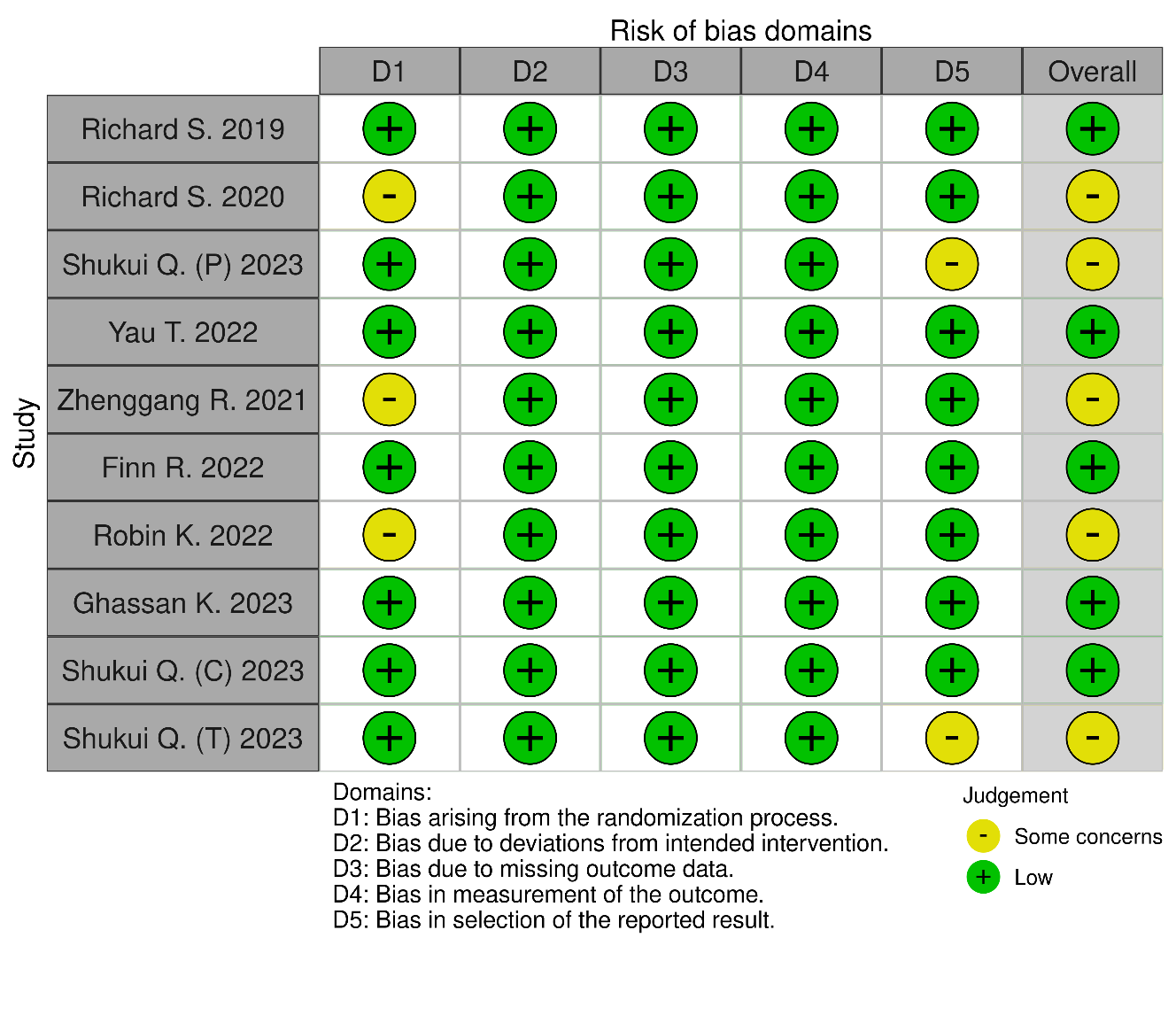


**Supplemental figure 2, subgroup analysis of ECOG=0 in PFS.**


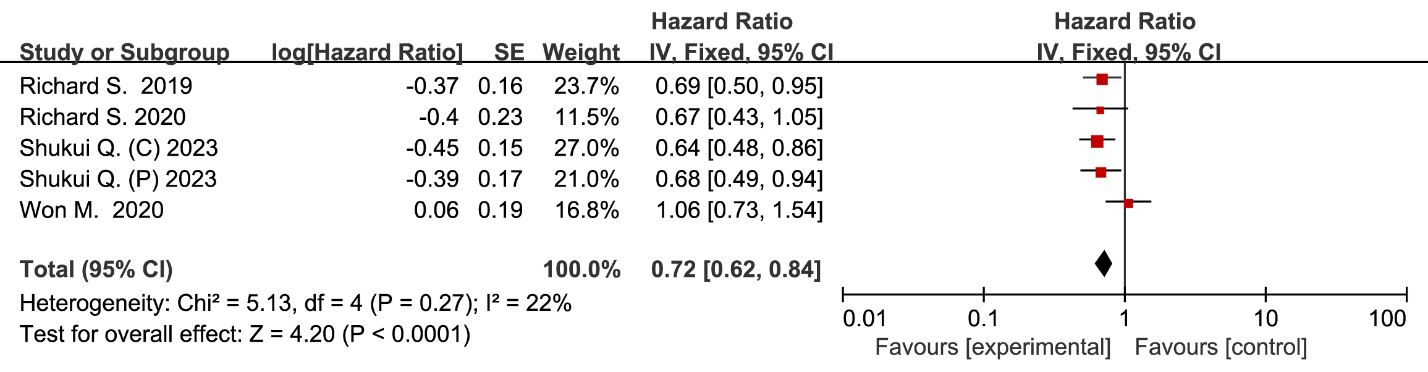


**Supplemental figure 3, subgroup analysis of ECOG≥1 in PFS.**


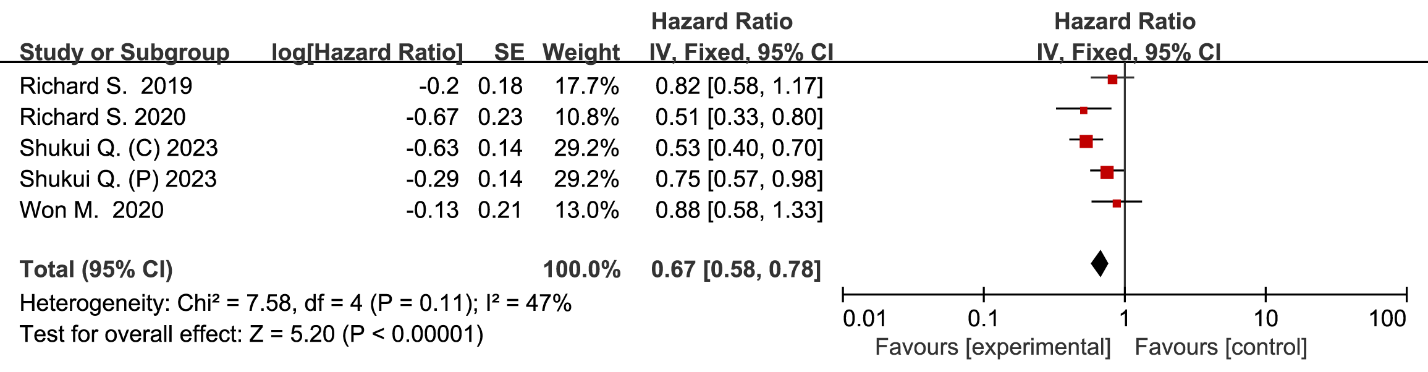


**Supplemental figure 4, subgroup analysis of Male in PFS.**


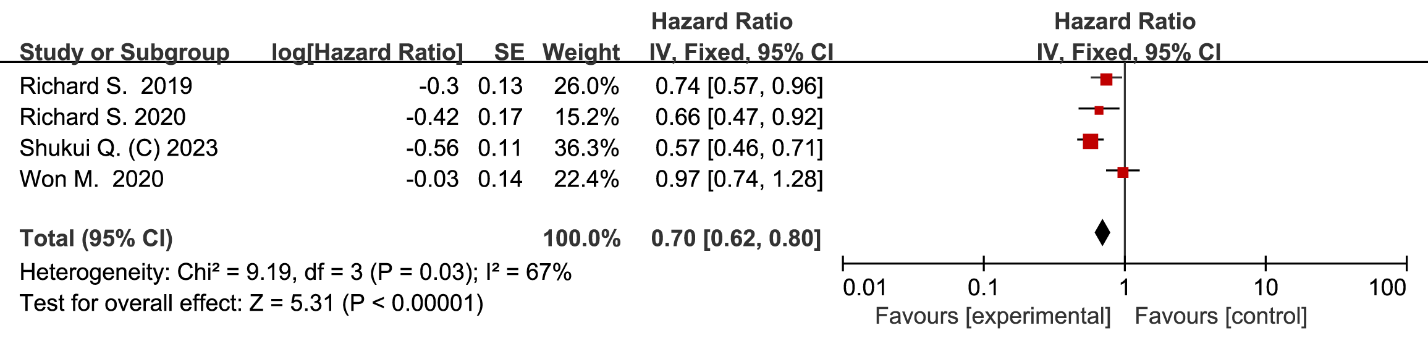


**Supplemental figure 5, subgroup analysis of Female in PFS.**


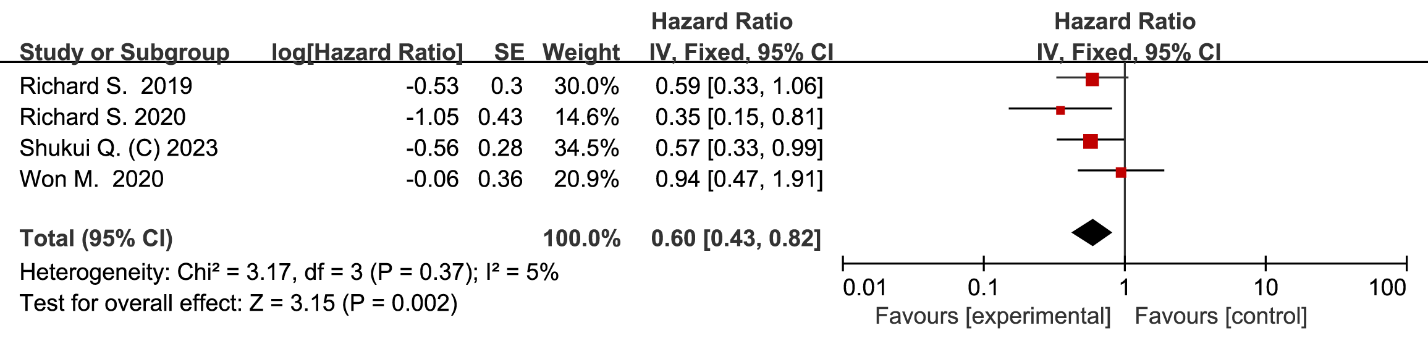


**Supplemental figure 6, subgroup analysis of HBV positive in PFS.**


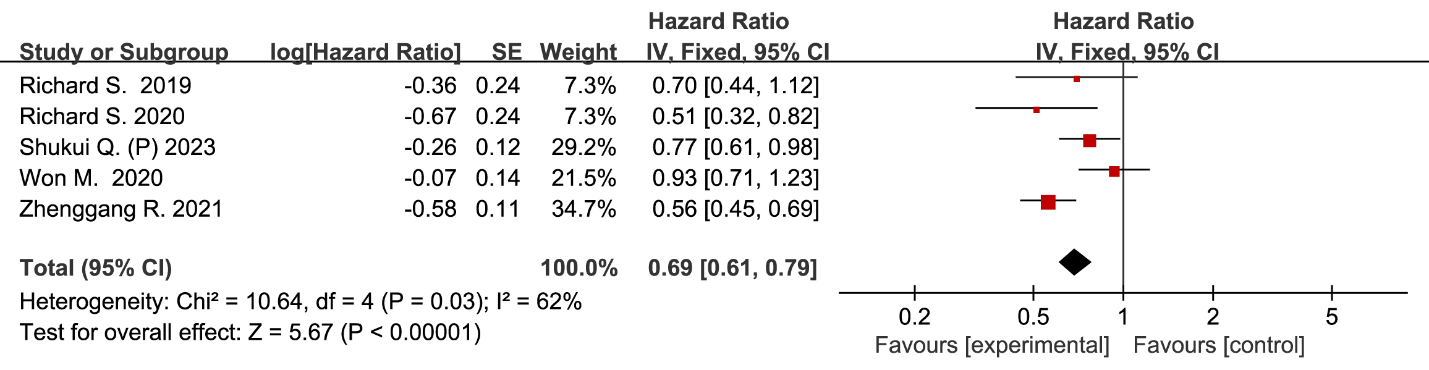


**Supplemental figure 7, subgroup analysis of HBV negative in PFS.**


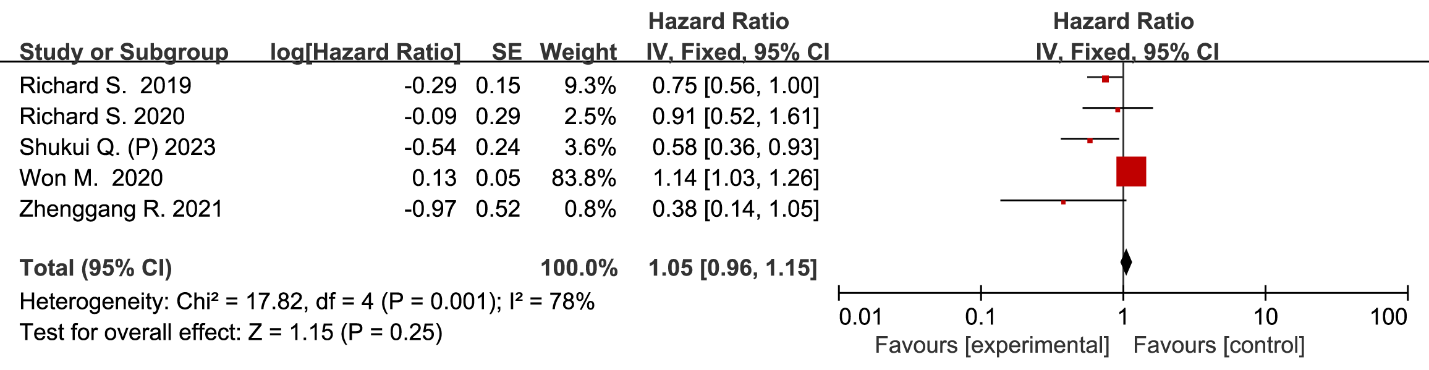


**Supplemental figure 8, subgroup analysis of macrovascular invasion positive in PFS.**


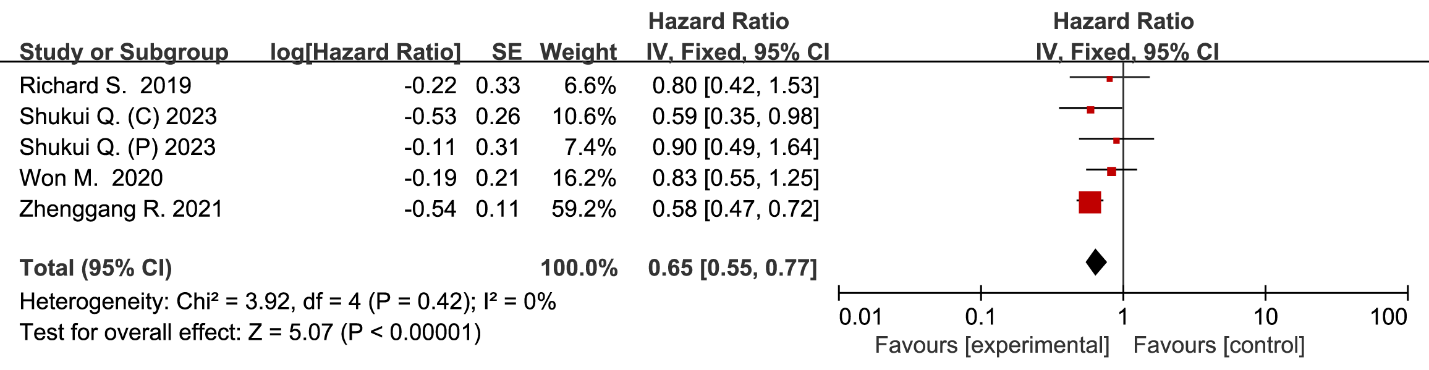


**Supplemental figure 9, subgroup analysis of macrovascular invasion negative in PFS.**


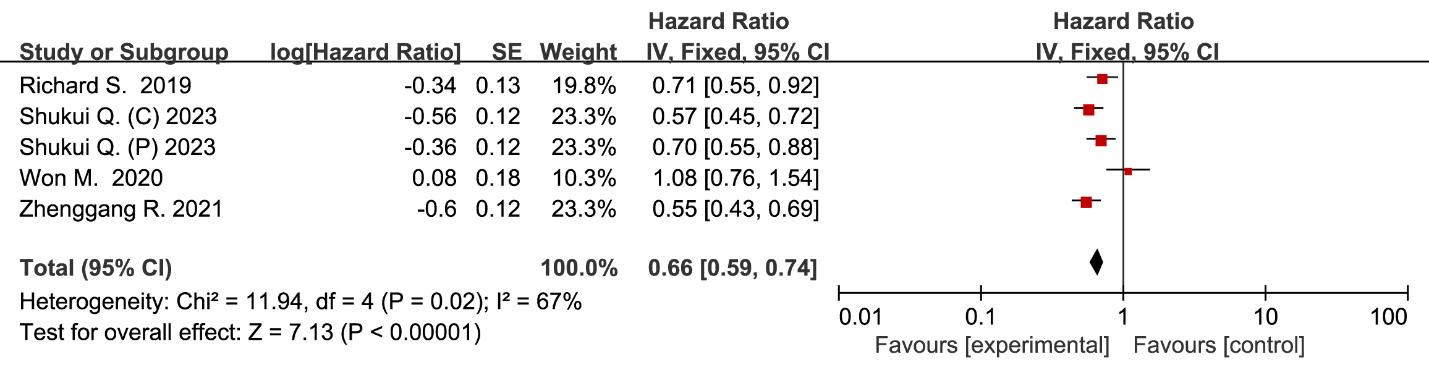


**Supplemental figure 10, publication bias and sensitivity analysis of** **ORR (A. Publication bias, *P* = 0.0042. B. Sensitivity).**


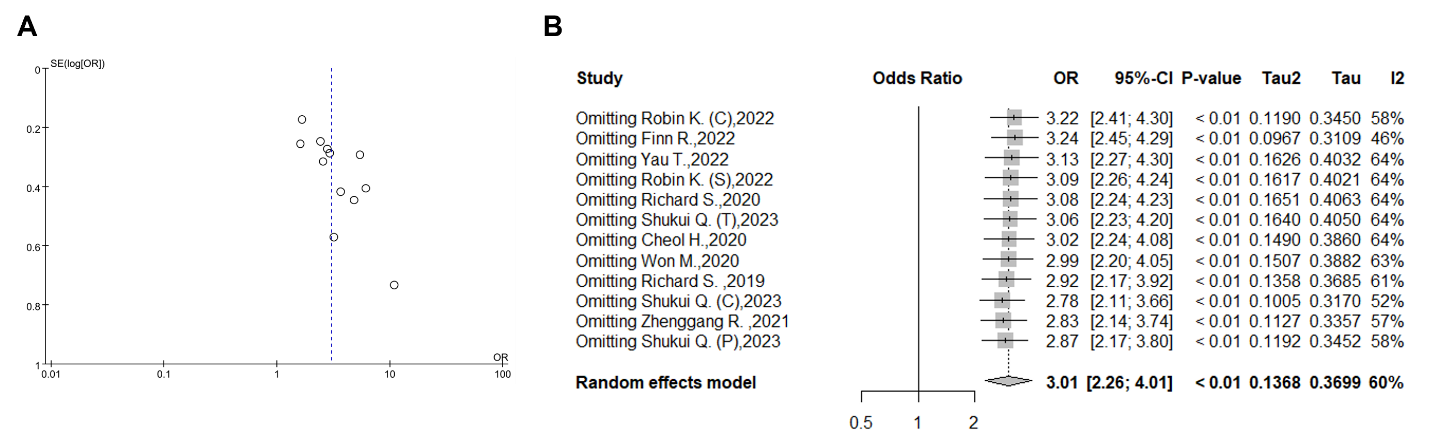


**Supplemental figure 11, publication bias and sensitivity analysis of DCR (A. Publication bias, *P* = 0.6625. B. Sensitivity).**


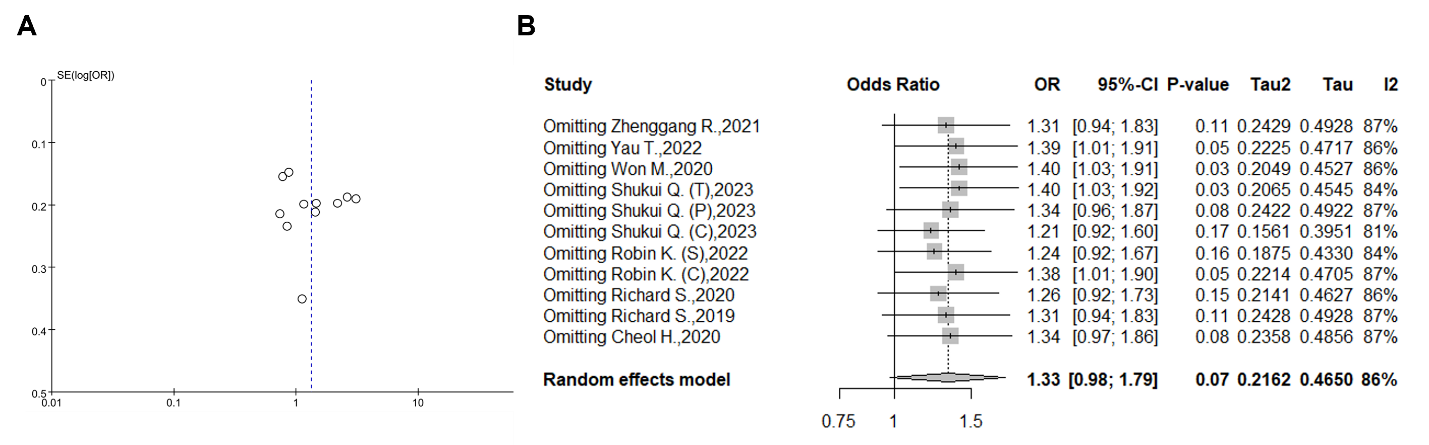


**Supplemental figure 12, publication bias and sensitivity analysis of SD (A. Publication bias, *P* = 0.8670. B. Sensitivity).**


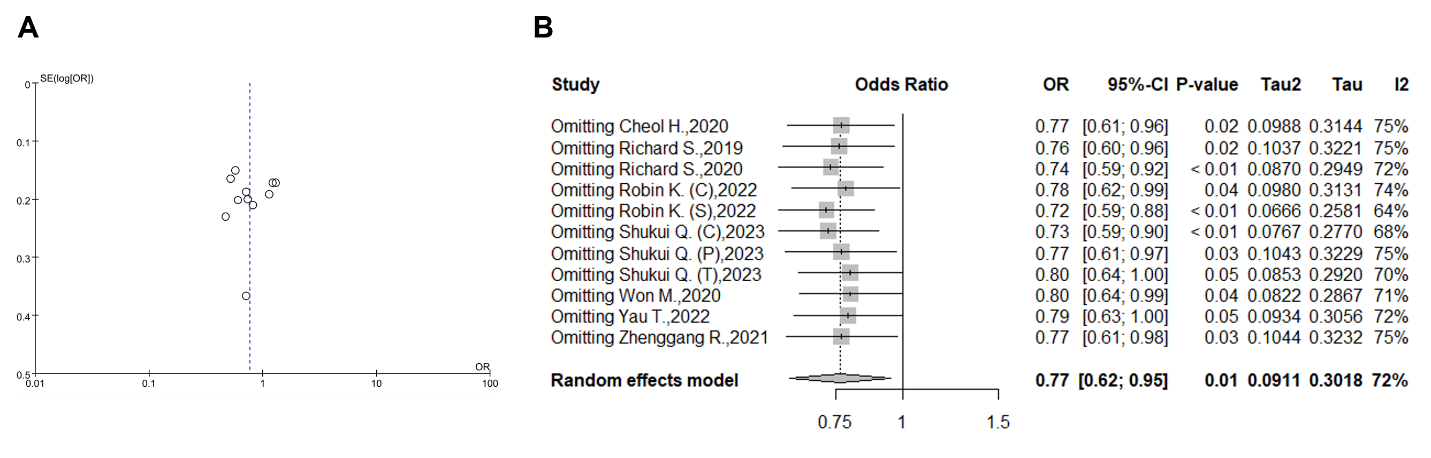


**Supplemental figure 13, publication bias and sensitivity analysis of PD (A. Publication bias, *P* = 0.2652. B. Sensitivity).**


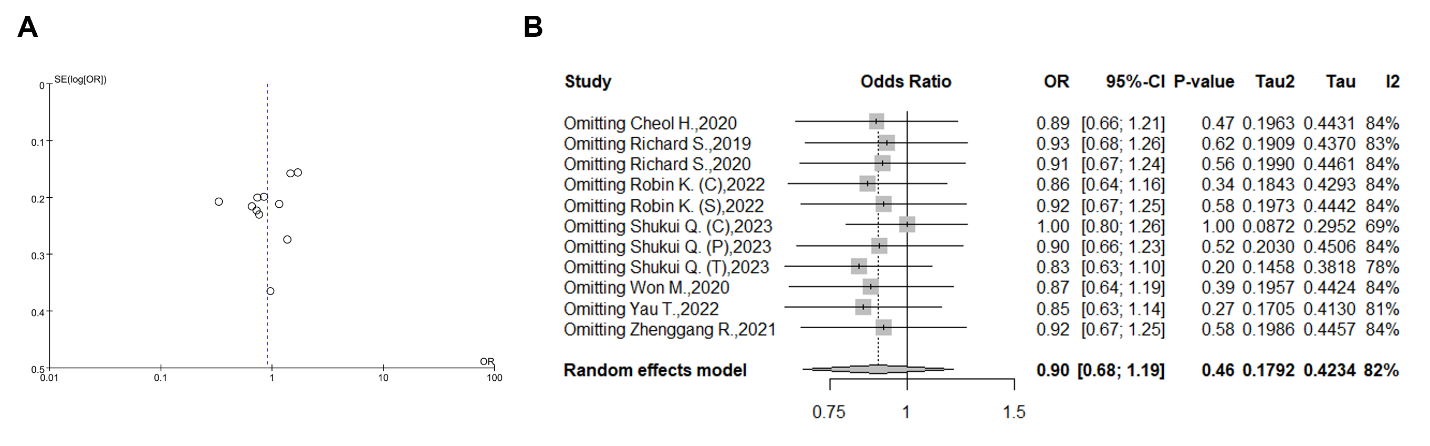


**Supplemental figure 14, publication bias and sensitivity analysis of** **any grade adverse events (A. Publication bias, *P* = 0.4690. B. Sensitivity).**


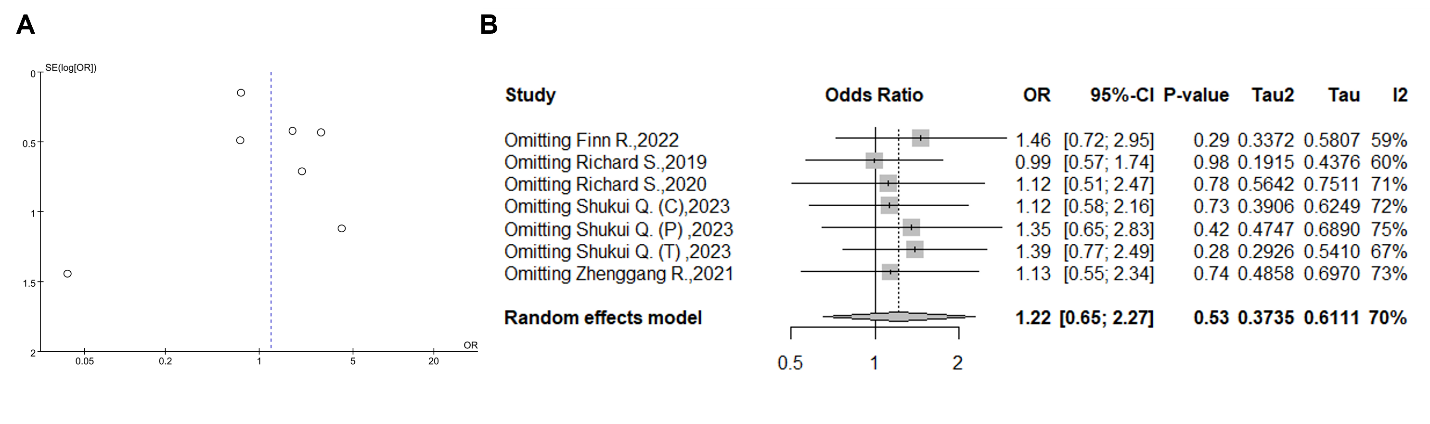


**Supplemental figure 15, publication bias and sensitivity analysis of** **all caused ≥grade 3 adverse events (A. Publication bias, *P* = 0.2126. B. Sensitivity).**


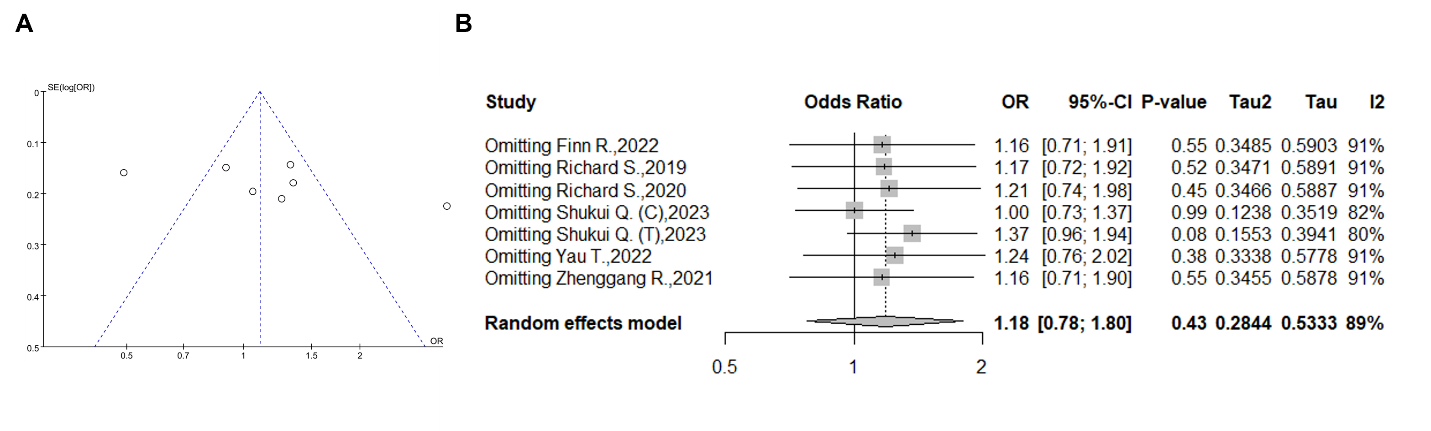


**Supplemental figure 16, publication bias and sensitivity analysis of** **treatment-related any grade adverse events (A. Publication bias, *P* = 0.4524. B. Sensitivity).**


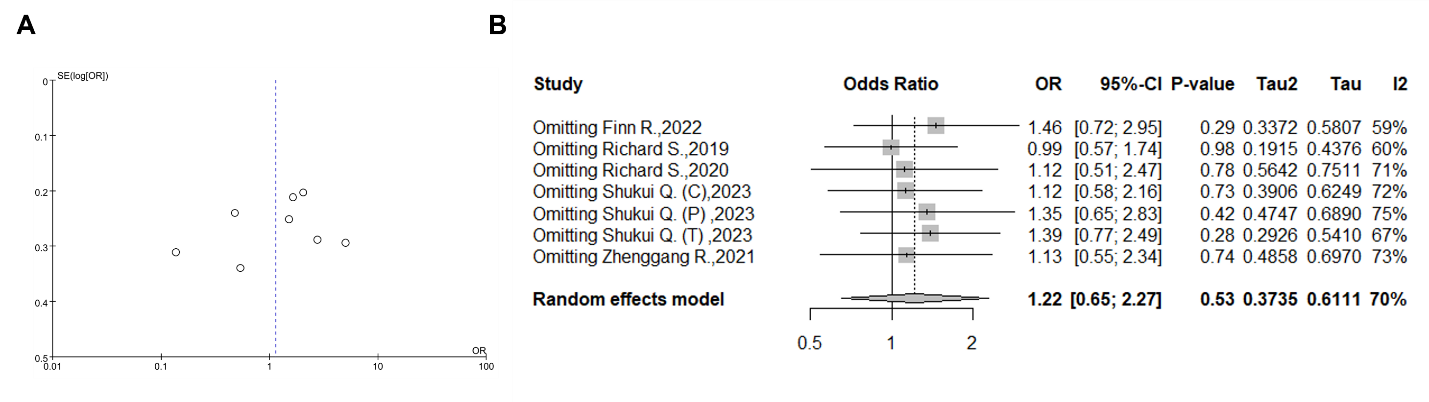


**Supplemental figure 17, sensitivity analysis of treatment-related ≥grade 3 events.**


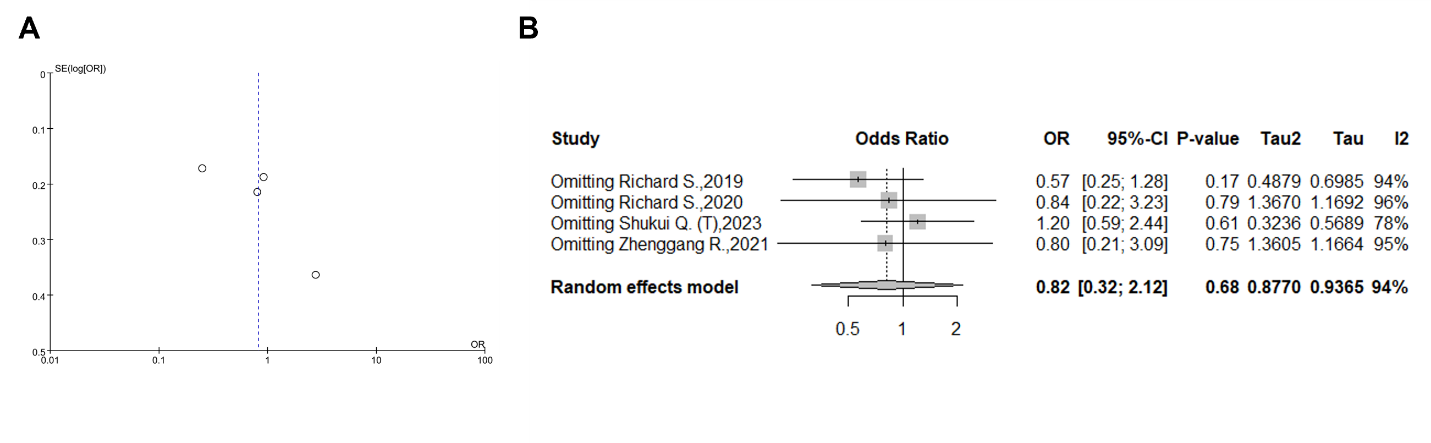


**Supplemental figure 18, sensitivity analysis of** **OS (A. Publication bias, *P* = 0.0058. B. Sensitivity of all studies. C. Sensitivity of subgroup).**


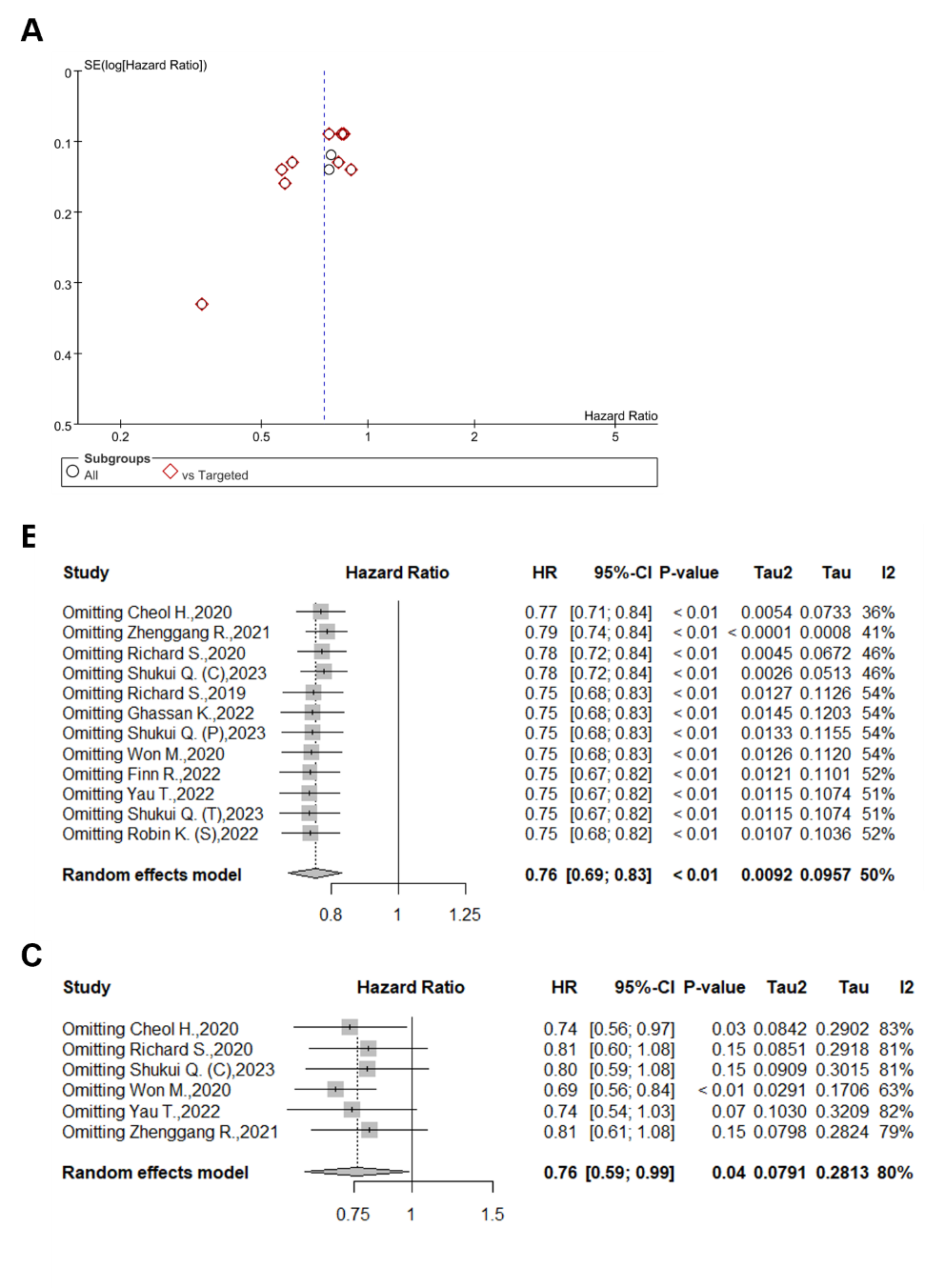


**Supplemental figure 19, sensitivity analysis of** **PFS (A. Publication bias, *P* = 0.3179. B. Sensitivity of all studies. C. Sensitivity of subgroup).**


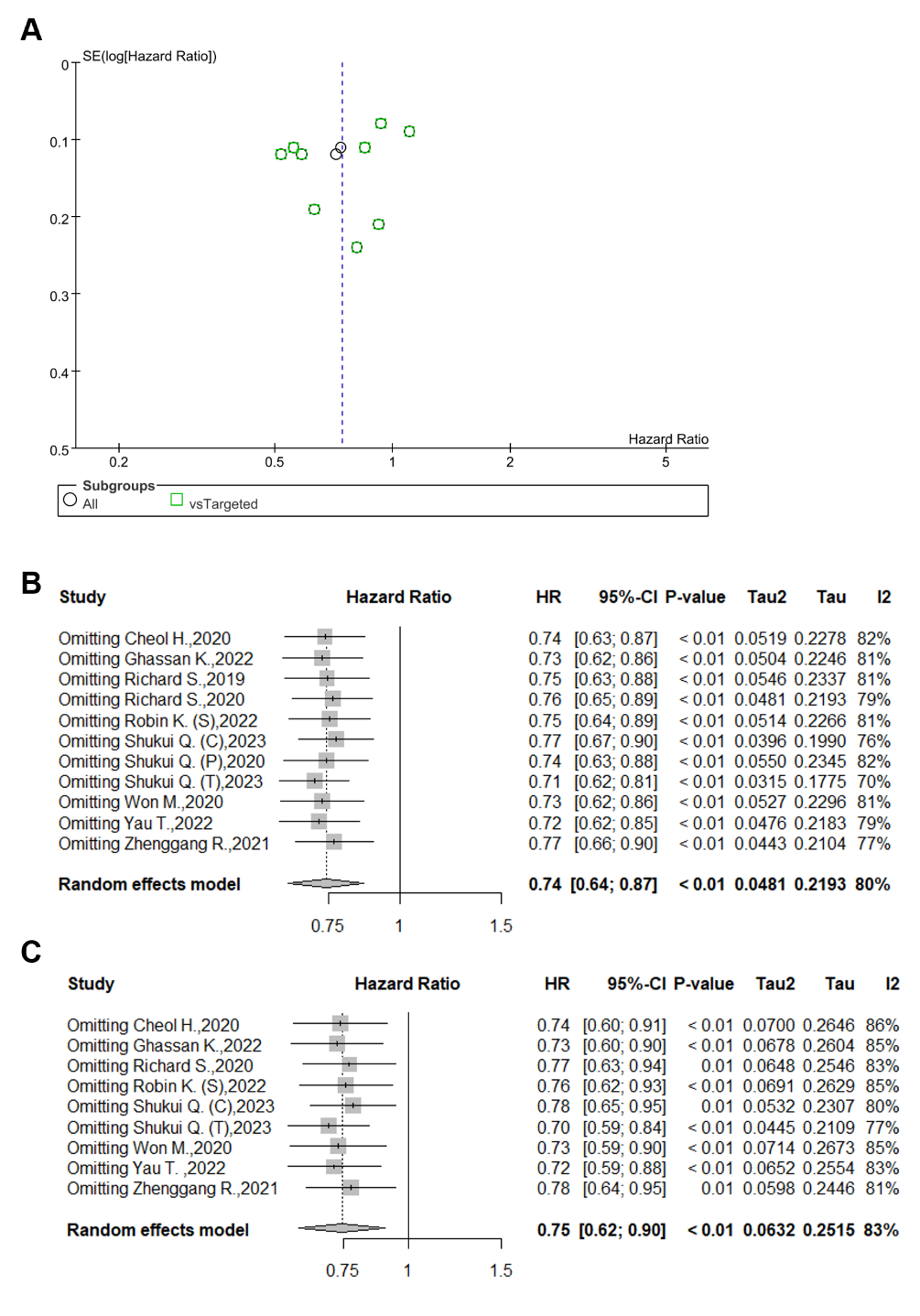

Supplement: Supplementary file 1 — Supplementery file1. Table S1. Search terms. Table S2. Published meta-analysis of ICIs treatment of HCC. Table S3. GRADE evidence assessment. Figure S1. The risk of bias. Figure S2. subgroup analysis of ECOG = 0 in PFS. Figure S3. subgroup analysis of ECOG ≥ 1 in PFS. Figure S4, subgroup analysis of male in PFS. Figure S5. subgroup analysis of female in PFS. Figure S6, subgroup analysis of HBV positive in PFS. Figure S7. subgroup analysis of HBV negative in PFS. Figure S8, subgroup analysis of macrovascular invasion positive in PFS. Figure S9. subgroup analysis of macrovascular invasion negative in PFS. Figure S10. publication bias and sensitivity analysis of ORR (A. Publication bias, P = 0.0042. B. Sensitivity). Figure S11. Publication bias and sensitivity analysis of DCR (A. Publication bias, P = 0.6625. B. Sensitivity). Figure S12. Publication bias and sensitivity analysis of SD (A. Publication bias, P = 0.8670. B. Sensitivity). Figure S13. Publication bias and sensitivity analysis of PD (A. Publication bias, P = 0.2652. B. Sensitivity). Figure S14. publication bias and sensitivity analysis of any-grade adverse events (A. Publication bias, P = 0.4690. B. Sensitivity). Figure S15. publication bias and sensitivity analysis of all caused ≥ grade 3 adverse events (A. Publication bias, P = 0.2126. B. Sensitivity). Figure S16. publication bias and sensitivity analysis of treatment-related any-grade adverse events (A. Publication bias, P = 0.4524. B. Sensitivity). Figure S17. sensitivity analysis of treatment-related ≥ grade 3 events. Figure S18, sensitivity analysis of OS (A. Publication bias, P = 0.0058. B. Sensitivity of all studies. C. Sensitivity of subgroup). Figure S19. sensitivity analysis of PFS (A. Publication bias, P = 0.3179. B. Sensitivity of all studies. C. Sensitivity of subgroup). [file 432_2023_5539_MOESM1_ESM.docx]
